# Oropharyngeal resistome remains stable during COVID-19 therapy, while fecal resistome shifts toward a less diverse resistotype

**DOI:** 10.1016/j.isci.2024.111319

**Published:** 2024-11-05

**Authors:** Elizaveta V. Starikova, Yulia S. Galeeva, Dmitry E. Fedorov, Elena V. Korneenko, Anna S. Speranskaya, Oksana V. Selezneva, Polina Y. Zoruk, Ksenia M. Klimina, Vladimir A. Veselovsky, Maxim D. Morozov, Daria I. Boldyreva, Evgenii I. Olekhnovich, Alexander I. Manolov, Alexander V. Pavlenko, Ivan E. Kozlov, Oleg O. Yanushevich, Natella I. Krikheli, Oleg V. Levchenko, Dmitry N. Andreev, Filipp S. Sokolov, Aleksey K. Fomenko, Mikhail K. Devkota, Nikolai G. Andreev, Andrey V. Zaborovsky, Sergei V. Tsaregorodtsev, Vladimir V. Evdokimov, Petr A. Bely, Igor V. Maev, Vadim M. Govorun, Elena N. Ilina

**Affiliations:** 1 Research Institute for Systems Biology and Medicine, Moscow 117246, Russian Federation; 2 Lopukhin Federal Research and Clinical Center of Physical-Chemical Medicine of Federal Medical Biological Agency, Moscow 119435, Russian Federation; 3 Moscow State University of Medicine and Dentistry, Moscow 127473, Russian Federation

**Keywords:** Clinical microbiology, Evolutionary mechanisms, Microbial genetics, Microbiome

## Abstract

Antimicrobial resistance poses a serious threat to global public health. The COVID-19 pandemic underscored the need to monitor the dissemination of antimicrobial resistance genes and understand the mechanisms driving this process. In this study, we analyzed changes to the oropharyngeal and fecal resistomes of patients with COVID-19 undergoing therapy in a hospital setting. A targeted sequencing panel of 4,937 resistance genes was used to comprehensively characterize resistomes. Our results demonstrated that the oropharyngeal resistome is homogeneous, showing low variability over time. In contrast, fecal samples clustered into two distinct resistotypes that were only partially related to enterotypes. Approximately half of the patients changed their resistotype within a week of therapy, with the majority transitioning to a less diverse and *ermB*-dominated resistotype 2. Common macrolide resistance genes were identified in over 80% of both oropharyngeal and fecal samples, likely originating from streptococci. Our findings suggest that the fecal resistome is a dynamic system that can exist in certain “states” and is capable of transitioning from one state to another. To date, this is the first study to comprehensively describe the oropharyngeal resistome and its variability over time, and one of the first studies to demonstrate the temporal dynamics of the fecal resistotypes.

## Highlights

• Oropharyngeal resistome dynamics described for the first time
• Three “core” streptococcal AR genes identified in oropharyngeal and fecal samples
• Two distinct resistotypes identified in fecal samples
• Half of the patients underwent resistotype shifts during COVID-19 therapy

## Graphical abstract

**Figure:**
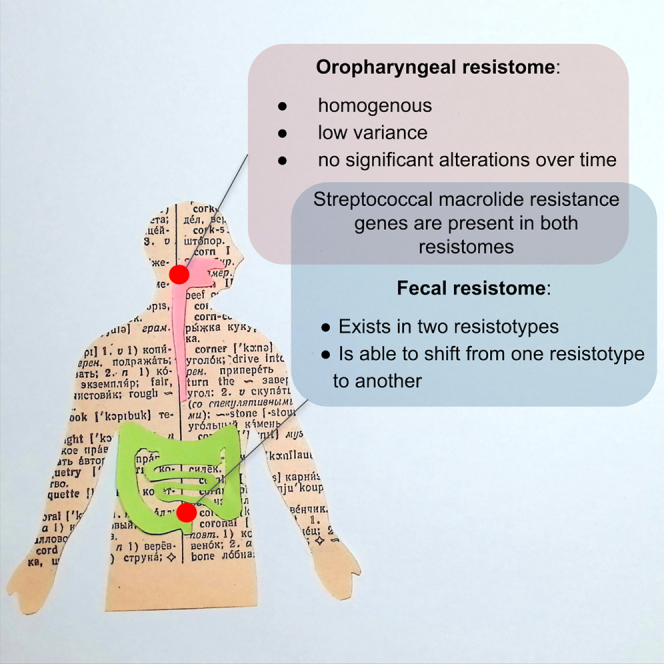

## Introduction

Antimicrobial resistance is a growing global health concern. The emergence of multidrug-resistant pathogens has an adverse effect on the efficacy of treatment for bacterial infections. The COVID-19 pandemic has underscored the importance of studying resistomes, as bacterial coinfections are common during viral respiratory illnesses and can significantly worsen outcomes. Microbial communities in the upper respiratory tract may be a potential source of such coinfecting drug-resistant bacteria. Pathogens such as pneumococci and staphylococci can reside in the nasal passages and the pharynx without causing any symptoms.^1^ In some cases, these pathogens can ascend to the lungs and cause infections in individuals with weakened immune systems. Some researchers posit that the microbial composition of the upper respiratory tract may also influence the course of SARS-CoV-2 infection, with respiratory bacteria potentially interfering with viral particle binding mechanisms.^2^^,^^3^

At the same time, the gut microbiome was hypothesized to serve as a reservoir for antimicrobial resistance (AR) genes, which can be conveyed to pathogenic bacteria via horizontal gene transfer.^4^ Once mobilized by transposons or plasmids, AR genes can be spread across different bacterial species, genera, and even families.^5^

Studying resistomes comes with challenges, as microbial communities can contain thousands of different sequences associated with antibiotic resistance. Shotgun metagenomic sequencing has its advantages, however, AR genes only represent a small fraction of metagenomic sequences, and the results of the resistome analysis are highly dependent on the sequencing depth of the samples. This makes it challenging to access the true diversity of AR genes in a microbial community and complicates the comparison of different samples. Moreover, the analysis of oropharyngeal samples presents additional challenges, including the presence of a significant amount of human DNA that cannot be easily removed. Targeted gene panel sequencing has previously been shown to be a reliable method for specifically capturing resistomes and has shown better sensitivity than shotgun metagenomic sequencing.^6^

In this study, we evaluated changes in the oropharyngeal and fecal resistomes of patients undergoing COVID-19 therapy in a hospital setting. To assess the AR gene composition of the samples, we employed a newly designed targeted gene sequencing panel that allowed us to capture up to 4,937 AR determinants. Furthermore, we assessed the bacterial taxonomy composition of the same samples using 16S rRNA gene amplicon sequencing.

We deliberately did not include healthy controls as our focus was on resistome dynamics throughout the course of the disease. This approach allowed us to track of even small differences in gene abundance that might be otherwise overlooked when comparing diseased and control groups. The Anna Karenina principle applied to human microbiomes suggests that all microbiomes that are considered “healthy” are similar in their composition, whereas each disease-associated microbiome has a unique pattern of “sickness.”^7^ Therefore, the resistomes of a cohort of patients diagnosed with the same disease and sampled at two time points were analyzed in order to track the changes that the patients’ resistomes and microbiomes underwent during therapy.

To our knowledge, this is the first study to compare the oropharyngeal and the fecal resistomes, and one of the first studies to analyze gut microbiome resistotypes in a dynamic manner.

## Results

### Samples and patients

**Figure 1:**
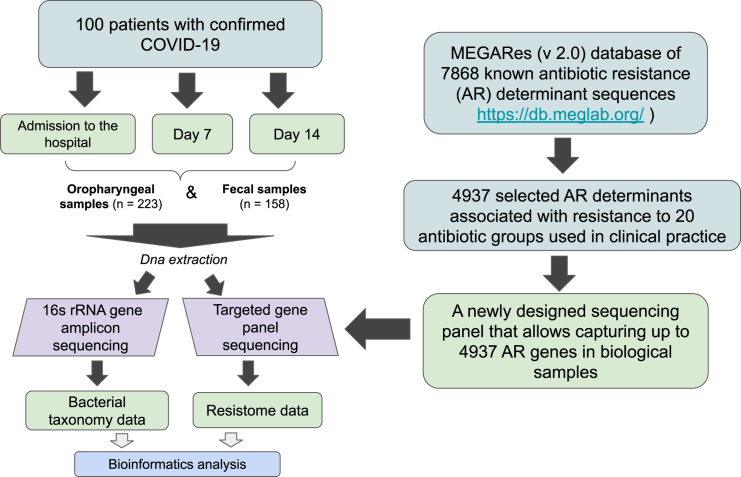
Experiment scheme

A total of one hundred patients with a confirmed SARS-CoV-2 infection who were undergoing therapy for the novel coronavirus disease (COVID-19) were enrolled in the study (see Figure 1 for the study design). The cohort consisted of 49 males and 51 females, with an age range between 25 and 88 years.

A total of 223 oropharyngeal and 158 fecal samples were collected from 100 patients at the Clinical Medical Center “Kuskovo” of the Moscow State University of Medicine and Dentistry (MSUMD) between April and June 2021. The majority of samples were collected at two time points: at the time of admission to the hospital and seven days after admission. For some of the patients, it was possible to collect samples on the fourteenth day after admission to the hospital (see Tables 1 and 2).

**Table 1:** Summary of samples collected from patients with COVID-19 in a hospital setting

Oropharyngeal samples (*n* = 223)			Fecal samples (*n* = 158)		
Admission	7 days	14 days	Admission	7 days	14 days
*n* = 95	*n* = 92	*n* = 36	*n* = 82	*n* = 62	*n* = 14

**Table 2:** Summary of patients who were sampled at different time points

Oropharyngeal samples			Fecal samples			Total patients
Admission	7 days	14 days	Admission	7 days	14 days	
X	–	–	–	–	–	*n* = 95
X	X	–	–	–	–	*n* = 91
X	X	X	–	–	–	*n* = 32
–	–	–	X	–	–	*n* = 82
–	–	–	X	X	–	*n* = 51
–	–	–	X	X	X	*n* = 6
X	–	–	X	–	–	*n* = 77
X	X	–	X	X	–	*n* = 45
X	X	X	X	X	X	*n* = 5

For the full list of the patients and the collected samples, see Table S1.

The standard treatment plans included glucocorticoids, such as dexamethasone; mucolytics, such as acetylcysteine; anticoagulants, such as enoxaparin sodium, atorvastatin, or heparin; and proton pump inhibitors (PPIs), such as omeprazole. Some patients were also administered antibiotics, such as cephalosporins, penicillins, or macrolides. Of the 100 patients sampled in the current study, 96 recovered, and four died.

### Targeted antimicrobial resistance gene panel design and sequencing

We have designed a targeted antimicrobial resistance (AR) gene sequencing panel based on 4,937 nucleotide sequences selected from the MEGARes (v.2.0) database.^8^ The sequences correspond to different variants of 646 AR genes that were responsible for specific resistance to 20 antibiotic types as well as nonspecific resistance (such as multidrug efflux pumps). A summary of the sequences corresponding to specific antibiotic types and resistance mechanisms can be found in Tables S2 and S3. We did not include AR genes responsible for tetracycline and aminoglycoside resistance, as previous researchers reported that these genes were present in all the fecal samples analyzed.^9^ Our objective, therefore, was to describe the distribution of other AR genes in patients’ microbiomes.

The complete set of nucleotide sequences used for targeted gene sequencing panel design can be downloaded at https://figshare.com/articles/dataset/4937_AR_determinant_sequences_from_MEGARes/23744580.

Based on the aforementioned sequences, a total of 5,277 probes were designed, including: 4,621 sequences covered by a single probe, 294 sequences covered by two probes, 20 sequences covered by three probes, and 2 sequences covered by four probes (see Table S4). High-throughput sequencing was conducted on the MGI DNBSEQ-G400 platform (2 × 150 bp paired-end sequencing). The median number of sequencing reads obtained per sample was 648,161.

The sequencing reads for resistome sequencing data have been deposited in the NCBI BioProject database under the accession number PRJNA1005621.

### Antimicrobial resistance gene panel data processing and analysis

#### Core resistome

The AR genes and AR gene sequence variants present in at least 80% of samples were considered “core.” We have identified seven “core” genes in each biotope that are listed in Table 3. Some of these genes were present in specific “core” variants (see Tables S5 and S6). For example, a particular streptococcal variant of the *ermB* macrolide resistance gene and the *lnuC* lincosamide resistance gene were found in 91% and 87% of fecal samples, respectively. Many other genes displayed variability, being present in different sequence variants.

**Table 3:** Core AR genes identified in oropharyngeal and fecal samples

Fecal samples				Oropharyngeal samples			
Gene	Antibiotic type	N var^a^	% smp^b^	Gene	Antibiotic type	N var	% smp^b^
*ermB*	Macrolide	19	95	*ermB*	Macrolide	18	81
*mefA*	Macrolide	14	87	*mefA*	Macrolide	14	99
*msrD*	Macrolide	2	85	*msrD*	Macrolide	2	99
*catA*	Chloramphenicol	18	93	*cfx*	Beta-lactam	13	97
*ermF*	Macrolide	6	88	*lsaC*	Lincosamide, streptogramin	1	89
*lnuC*	Lincosamide	1	87	*patA*	Quinolone	1	81
*sat*	Nucleoside	4	80	*ermX*	Macrolide	7	80

a The number of known sequence variants of these genes identified in samples of a certain biotope.

b The percent of samples of a certain biotope having this gene in at least one sequence variant Also see Tables S5 and S6 for “core” AR gene sequence variants, and Table S7 for biotope-specific sequence variants.

Three “core” genes that intersect between the two biotopes have been identified: *ermB*, *mefA,* and *msrD* genes, which are associated with macrolide resistance. Our findings revealed the presence of “biotope-specific” sequence variants of the *ermB* and *mefA* genes, which were more prevalent in either fecal or oropharyngeal samples. These variants belonged to different bacterial species and genera. Therefore, one *mefA* variant and eight *ermB* variants were identified as being more prevalent in fecal samples, while two *mefA* variants were observed to be more common in oropharyngeal samples. The two oropharyngeal *mefA* sequence variants were reported in *Streptococcus* genomes, according to the nt database. Additionally, one fecal *mefA* sequence variant was reported in *Clostridium* genomes, although none of the species belonging to *Clostridium* genera were identified in fecal samples through 16S rRNA gene sequencing analysis. The eight fecal *ermB* sequence variants were identified in various bacterial genomes, including those of *Lactobacillus*, *Streptococcus*, *Enterococcus*, and other genera.

All “biotope-specific” sequence variants with their possible carriers are listed in Table S7.

At the same time, four genes were identified as being widespread within a single biotope. The “core” oropharyngeal AR genes included class A beta-lactamase *cfx*, class 2 ABC transporter *lsaC*, ABC efflux pump gene *patA,* and 23S rRNA methyltransferase *ermX*. The “core” fecal AR genes included chloramphenicol acetyltransferase *catA*, streptococcal lincosamide nucleotidyltransferase *lnuC*, streptothricin acetyltransferase *sat,* and 23S rRNA acetyltransferase *ermF*. The fecal *sat* and *ermF* genes were found in multiple sequence variants.

#### Top antimicrobial resistance genes by abundance

**Figure 2:**
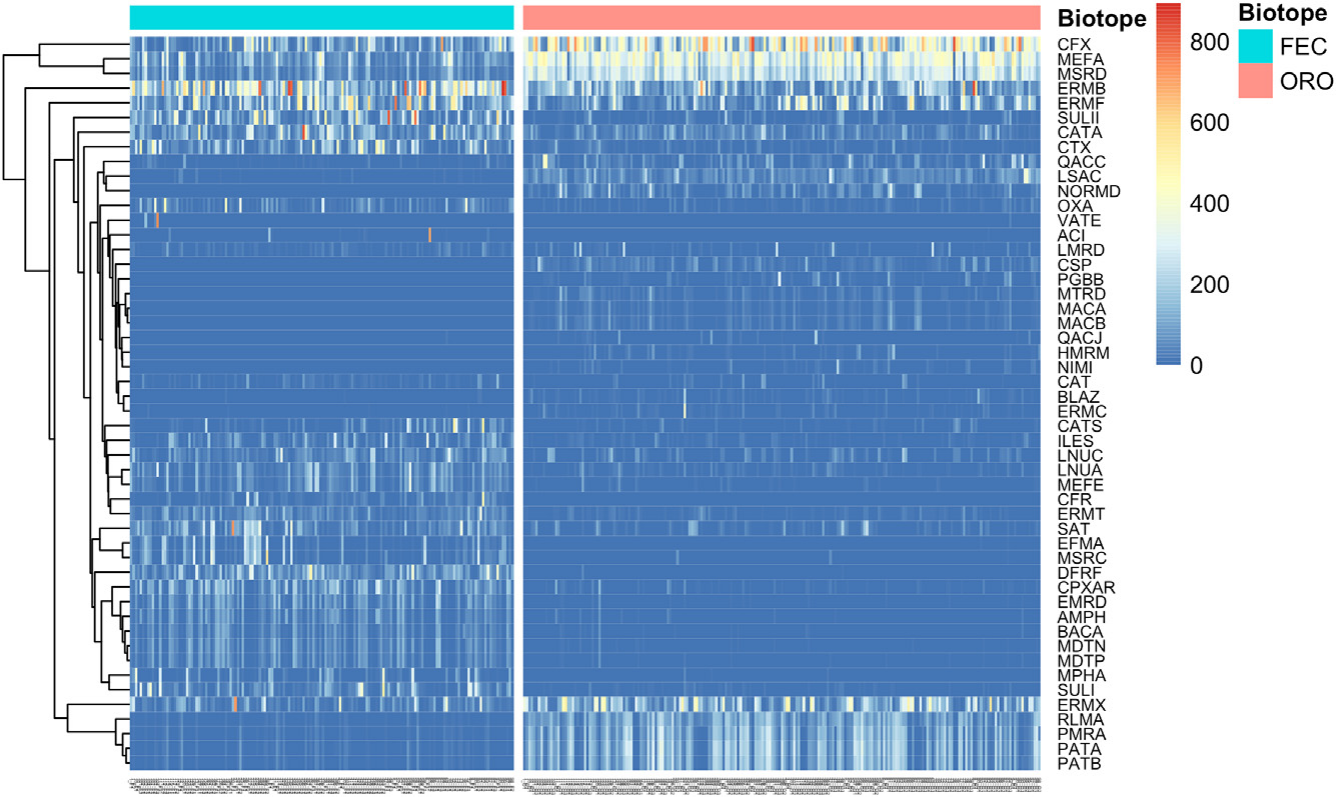
A heatmap comparing the most abundant AR genes of the fecal (FEC) and oropharyngeal (ORO) samples The heatmap is based on square-root transformed RPKM values. Fecal resistome (to the left, blue bar) is characterized with high abundances of *ermB* and *ermF* genes. Oropharyngeal resistome (to the right, pink bar) is characterized with high abundances of *cfx*, *mefA*, and *msrD* genes. X axis represents individual samples.

The most abundant AR genes for each biotope are shown in Figure 2. The macrolide resistance genes *ermB* and *ermF* exhibited the highest total abundance in fecal samples, as calculated using both RPKM and TPM metrics. Additionally, sulfonamide-resistant dihydropteroate synthase *sulII*, chloramphenicol acetyltransferase *catA*, and class A beta-lactamases *ctx* and *cfx* demonstrated high total abundance. Similarly, beta-lactamase *cfx* and macrolide resistance efflux pump genes *msrD* and *mefA* exhibited the highest abundance in oropharyngeal samples.

#### Resistome diversity

Resistome diversity was evaluated based on the number of genes that exhibited at least one sequence variant covered by reads at 99–100% of the gene length.

**Figure 3:**
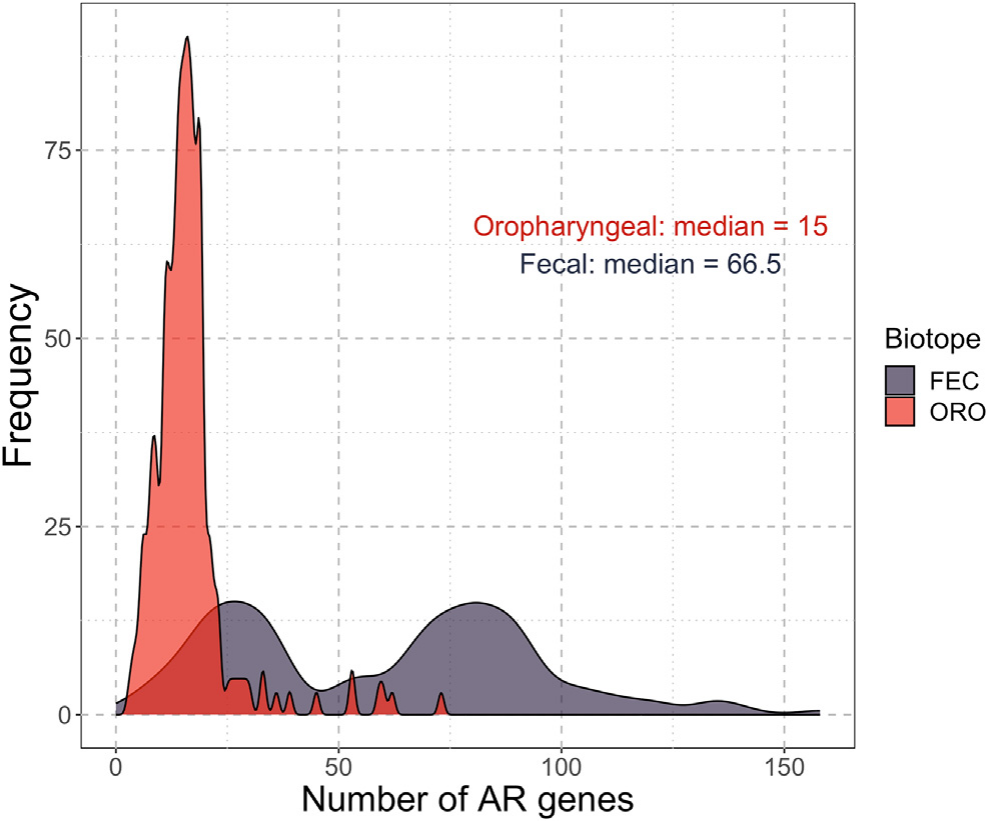
Distribution of the counts of antimicrobial resistance (AR) genes identified in each biotope Also see Figures S11 and S12 regarding the connection between the number of AR genes and Bray-Curtis distances between the sampled time points.

The number of AR genes identified in oropharyngeal samples ranged from 3 to 73 with a median of 15 genes and a standard deviation of 9.5 genes (see Figure 3). The number of AR genes identified in fecal samples ranged from 5 to 158 with a median of 66.5 genes and a standard deviation of 34 genes. A bimodal distribution of the number of AR genes identified in fecal samples was observed, which is explained later in discussion (see “Resistotypes” section).

#### Dissimilarity of fecal and oropharyngeal resistomes

**Figure 4:**
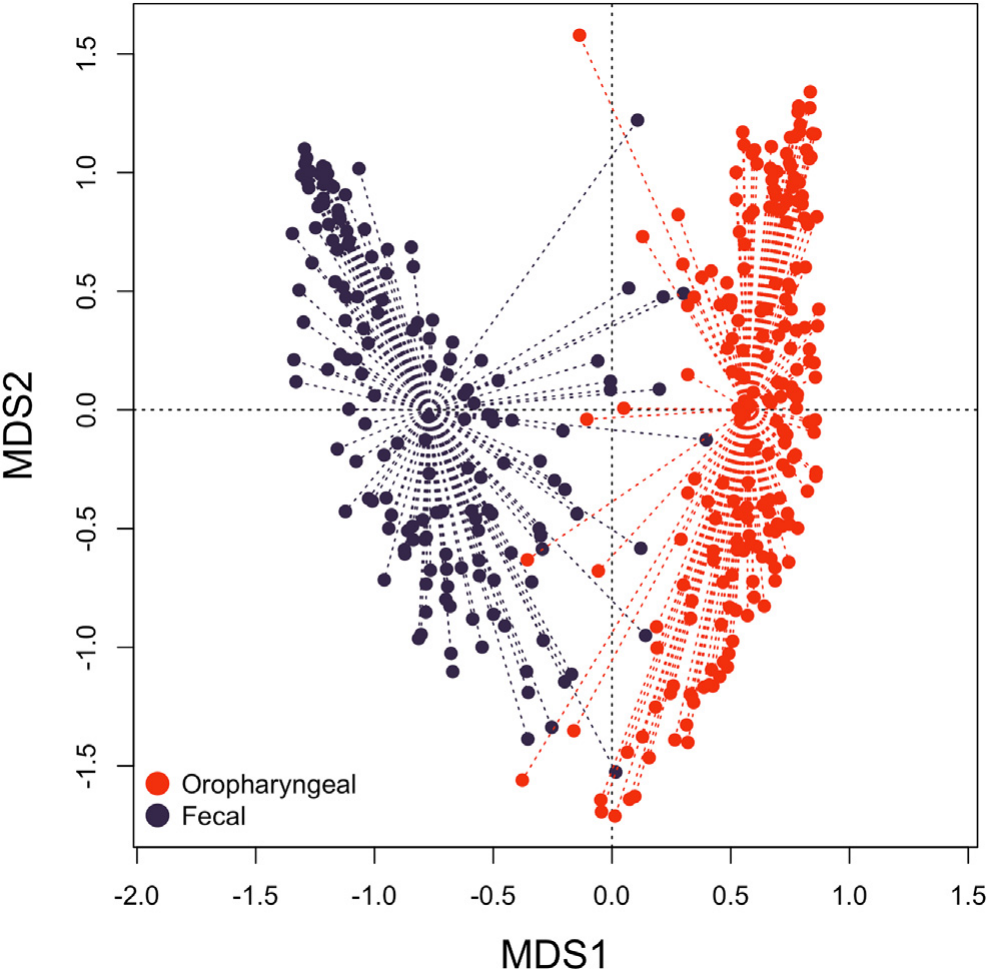
PCoA (principal coordinates analysis) plot based on Bray-Curtis distances between fecal and oropharyngeal samples Fecal samples are shown in dark blue. Oropharyngeal samples are shown in red. Samples from all time points are considered. Also see Table S8 for Bray-Curtis distances between paired samples.

To assess the dissimilarity of fecal and oropharyngeal resistomes, we calculated Bray-Curtis distances between all the samples from all the time points based on their RPKM values. A CAP (Canonical Analysis of Principal Coordinates) plot representing these dissimilarities is shown in Figure 4. The plot shows a clear partitioning of the oropharyngeal and fecal samples, with the exception of a few individual samples.

#### Sample dissimilarity within biotopes

To ascertain whether the resistome samples obtained from the same patient exhibit a greater degree of similarity to one another than to any other samples, we have assigned the sample with the lowest Bray-Curtis distance as the “closest” for each of the “paired” fecal and oropharyngeal samples. Of the 98 fecal samples, 18 (18.36%) demonstrated the highest similarity to their paired samples. Similarly, 13 out of the 168 (7.74%) oropharyngeal samples showed the highest similarity to their “paired” samples.

Bray-Curtis distances between the paired samples and minimal Bray-Curtis distances for each sample can be found in Table S8.

#### Resistotypes

**Figure 5:**
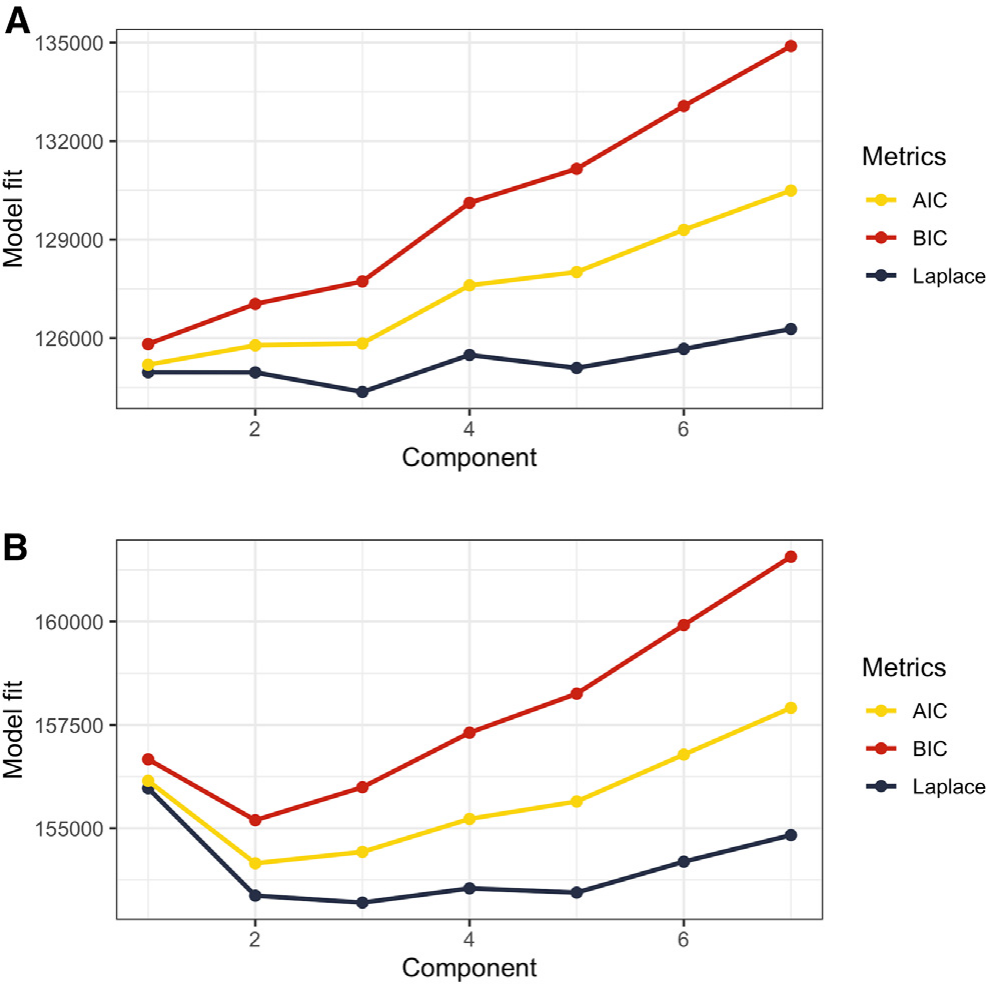
Model fit evaluation for Dirichlet Multinomial Mixture Models The analysis is performed based on RPKM values of: (A) Oropharyngeal samples (B) Fecal samples X axis displays the number of components, Y axis displays fit metrics: Bayesian Information Criterion (BIC), Akaike Information Criterion (AIC), and Laplace approximation. Lower metrics values indicate better model fit.

We attempted to identify potential resistotypes in both oropharyngeal and fecal samples using Dirichlet-multinomial mixture models. No discernible signs of clustering were identified in oropharyngeal samples (see Figure 5A). As for the fecal samples, all the three metrics used to evaluate model fit demonstrated a notable decline at k = 2 (see Figure 5B), indicating that the samples could be divided into two clusters, Resistotype 1 (RT1, *n* = 89 samples) and Resistotype 2 (RT2, *n* = 69 samples).

**Figure 6:**
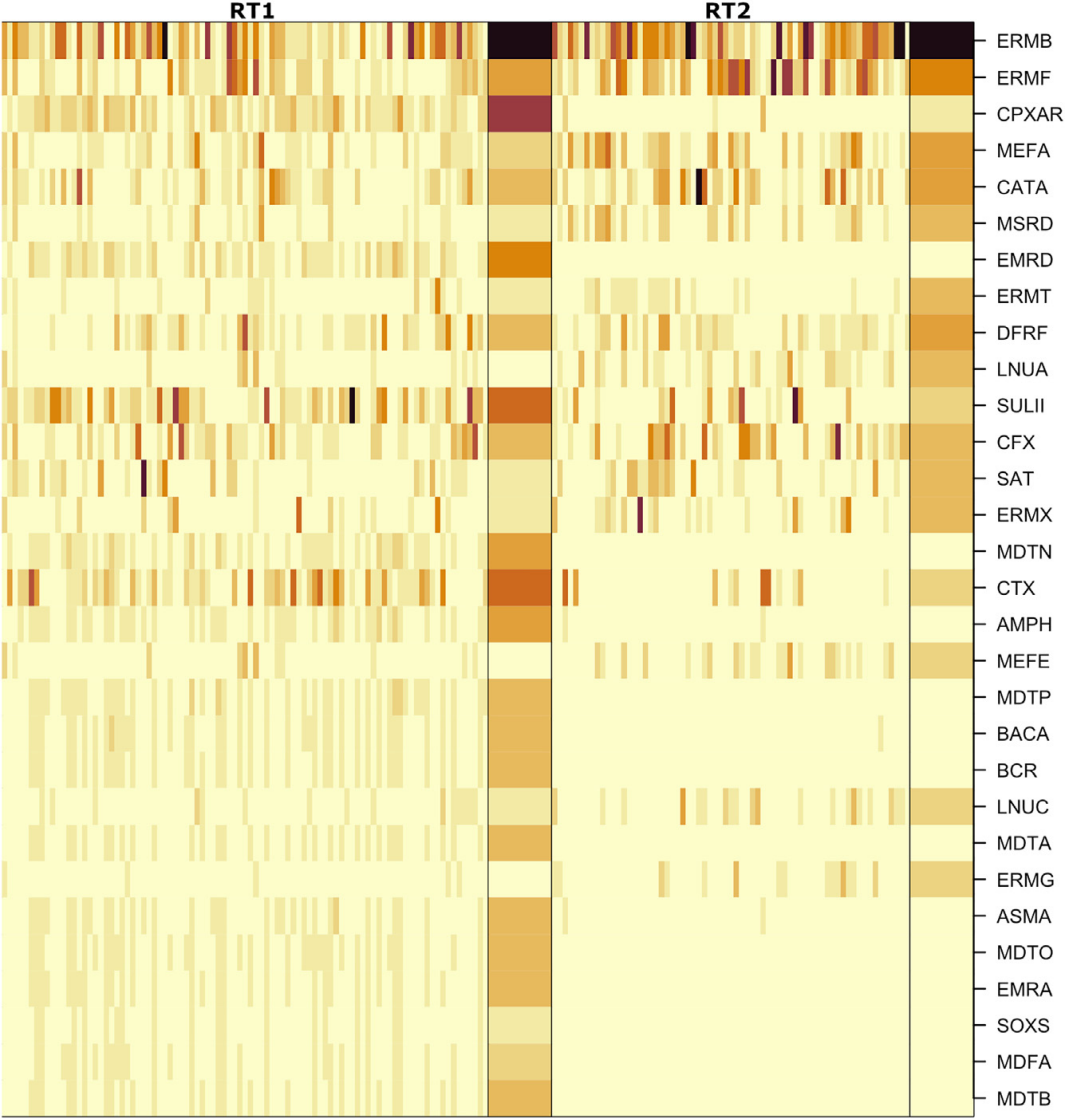
Heatmap showing the most abundant AR genes in fecal samples in the two identified fecal resistotypes The heatmap is based on square-root transformed RPKM values. Samples corresponding to resistotype RT1 are shown on the left, while samples corresponding to RT2 are shown on the right. Columns correspond to samples, and samples are grouped by Dirichlet component, with means summarized as a separate wide column to the right of each group. Darker colors correspond to higher RPKM values. Also see Figure S3 for resistotype drivers.

**Figure 7:**
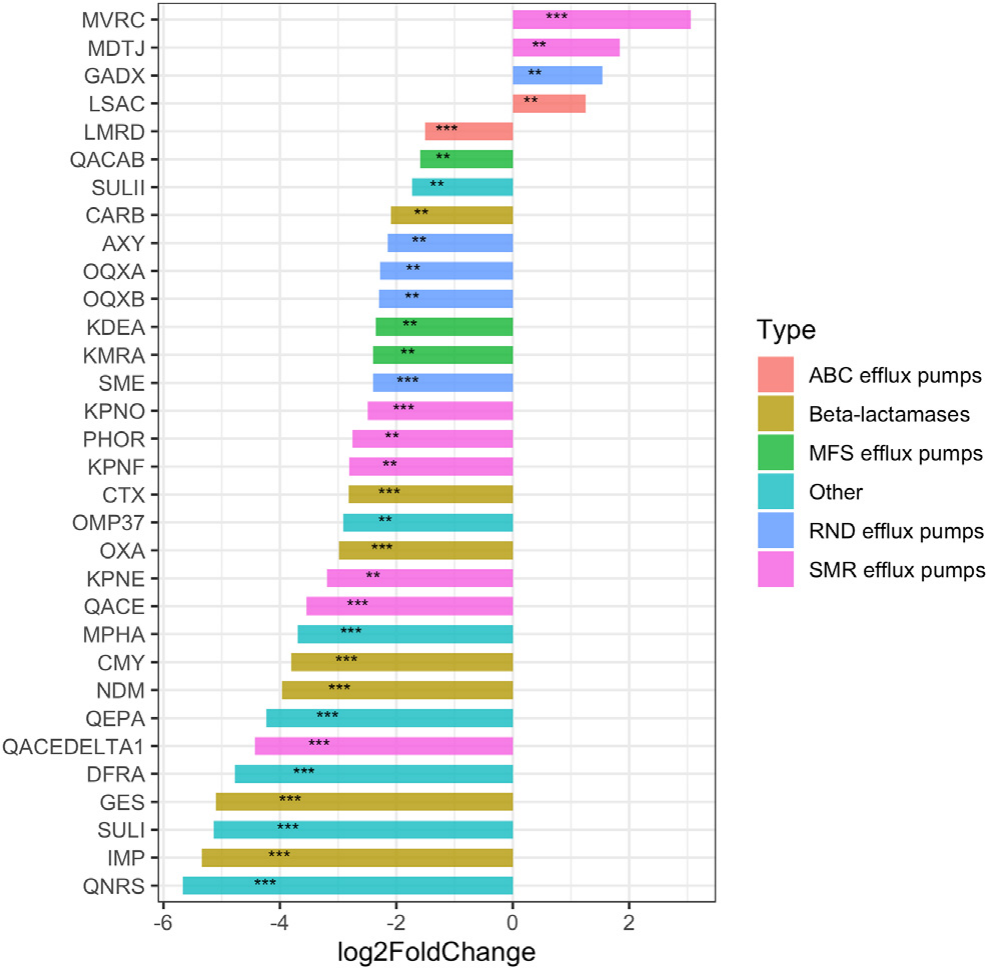
Barplots illustrating log2 fold change of AR gene counts between resistotypes 1 and 2 calculated during DESeq2 analysis Bars oriented right (positive log2FC) correspond to AR genes overrepresented in RT2, bars oriented left (negative log2FC) correspond to AR genes overrepresented in RT1. Genes with *p*-values < 0.05 are marked with “∗∗,” and species with *p*-values < 0.01 are marked with “∗∗∗.” Also see Table S10 for exact log2 fold change values, *p*-values and expected means.

Resistotype RT1 was characterized with *ermB*, *cpxar*, and *sulII* as the top drivers contributing to the cluster, and resistotype RT2 was mainly driven by *ermB* (see Figure 6 for a heatmap; Figure S3 for drivers; also see Figure 7 for the results of DESeq2 analysis).

**Figure 8:**
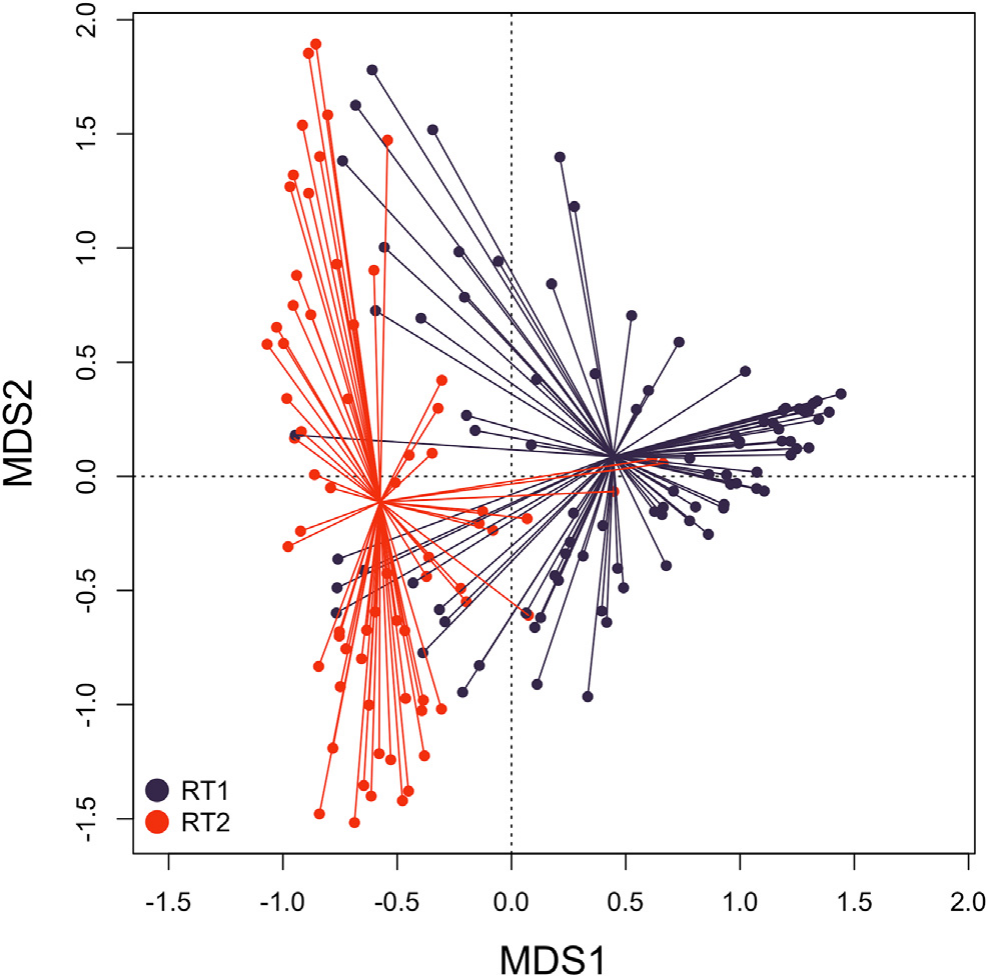
PCoA (principal coordinates analysis) plot based on Bray-Curtis between fecal samples assigned to resistotypes RT1 and RT2 RT1 samples are shown in dark blue, RT2 samples are shown in red. All time points are considered.

**Figure 9:**
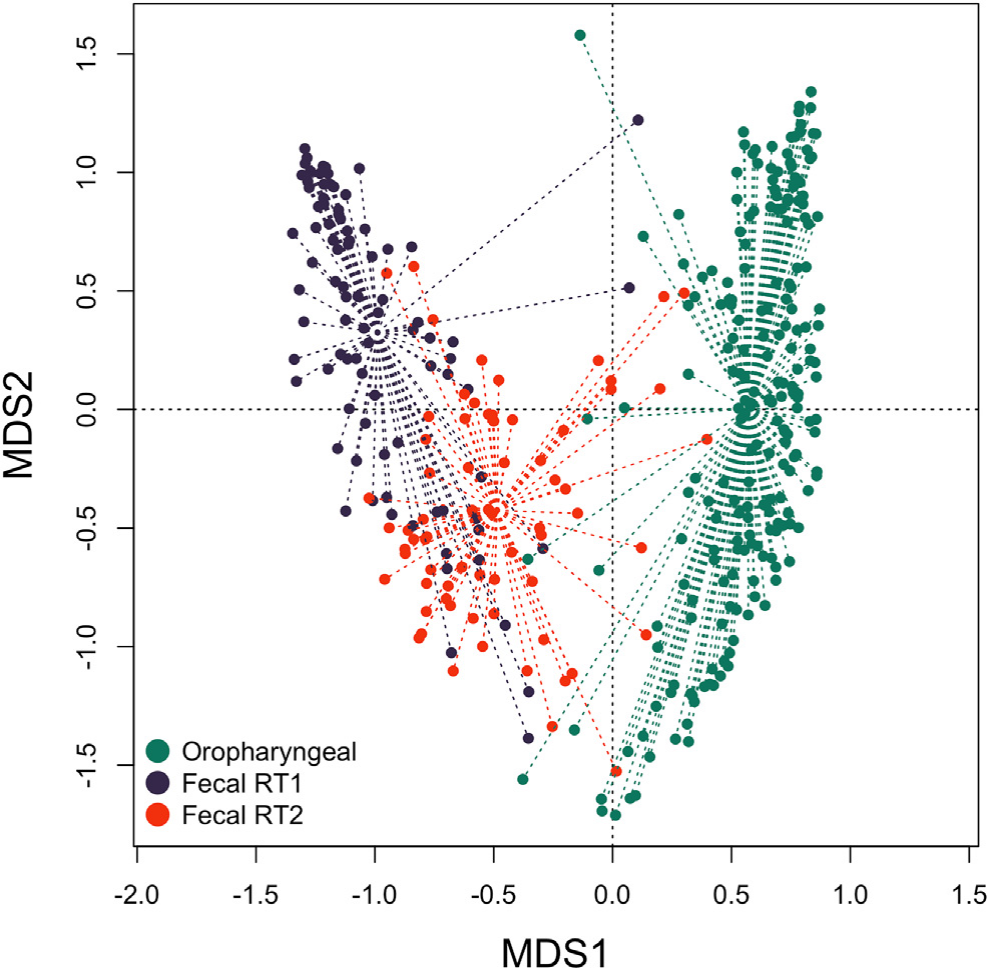
PCoA (Principal Coordinates Analysis) plot based on Bray-Curtis between oropharyngeal samples and fecal samples assigned to resistotypes RT1 and RT2 Oropharyngeal samples are shown in green, RT1 samples are is shown dark blue, and RT2 samples are shown in red. All time points are considered.

The dissimilarity of resistotypes RT1 and RT2 is shown in Figure 8. A principal coordinates analysis (PCoA) plot representing the Bray-Curtis distances of oropharyngeal samples and fecal samples belonging to RT1 and RT2 is presented in Figure 9. It can be seen on the latter plot that some samples belonging to fecal RT2 are more similar to oropharyngeal samples than those of fecal RT1.

To evaluate the reproducibility of the observed resistotypes, we have replicated the clustering 10 times, each with a different random number seed. In all iterations, the samples split into two clusters in the very same way, with the exception of one sample (087_F2) that was assigned to RT1 two times out of ten, and to RT2 eight times out of ten. To make the subsequent analysis simpler, we assigned RT2 to that sample, keeping in mind its intermediate state.

**Figure 10:**
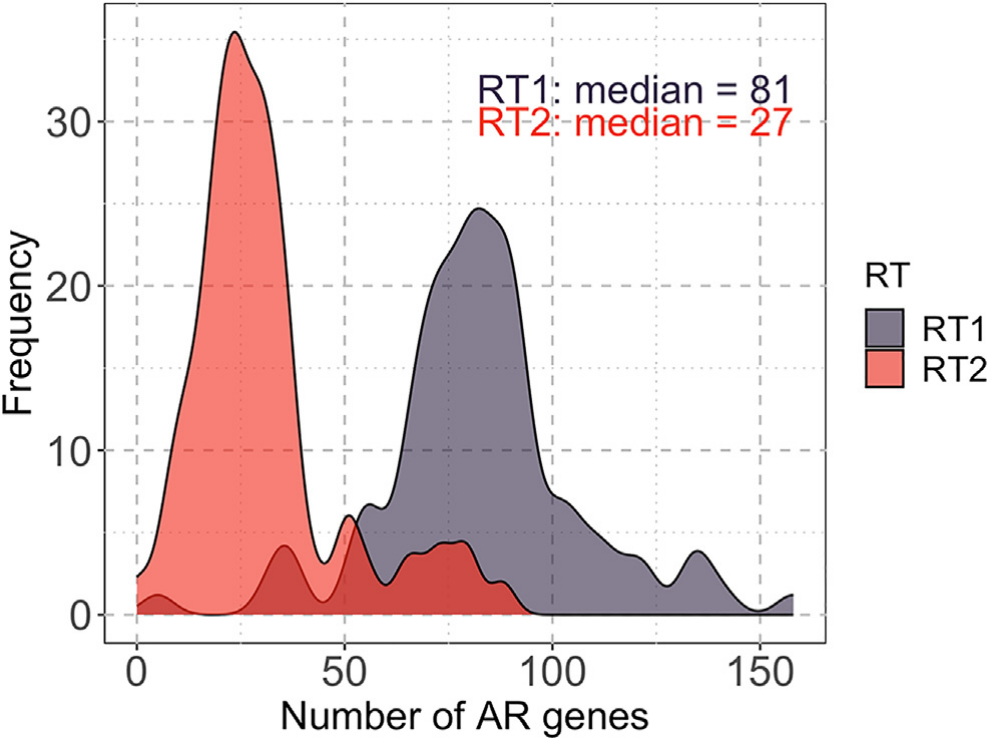
Distribution of the counts of antimicrobial resistance (AR) genes in each resistotype Also see Figure S4 that illustrates the distribution of the number of reads in samples belonging to each resistotype.

It is noteworthy that the samples belonging to RT1 and RT2 each showed a unimodal distribution of the numbers of AR genes identified (see Figure 10, which explains the bimodal distribution of the numbers of AR genes found in fecal samples that were discussed above (see “core resistome” subsection). RT1 showed a higher number of AR genes with a median of 81 genes and a standard deviation of 24 genes, while RT2 showed a lower number of AR genes with a median of 27 genes and a standard deviation of 14.5 genes.

It should be noted here that the assignment of samples to RT1 with a higher number of AR genes or to RT2 with a lower number of genes is unlikely to be explained by differences in sequencing depth, as shown in Figure S4.

It is also worth mentioning that, unlike AR gene diversity measures, bacterial alpha-diversity measures did not show a bimodal distribution in fecal samples. However, the Chao1 index obtained for bacterial taxonomy data was significantly higher in samples belonging to RT1 than in those belonging to RT2 (see Figure 19).

Interestingly, most of the samples (69%) assigned to RT1 were collected at “Day 0” time (upon admission to the hospital), while most of the samples (71%) assigned to RT2 were collected either one week (“Day 7”) or two weeks (“Day 14”) after admission to the hospital.

**Figure 11:**
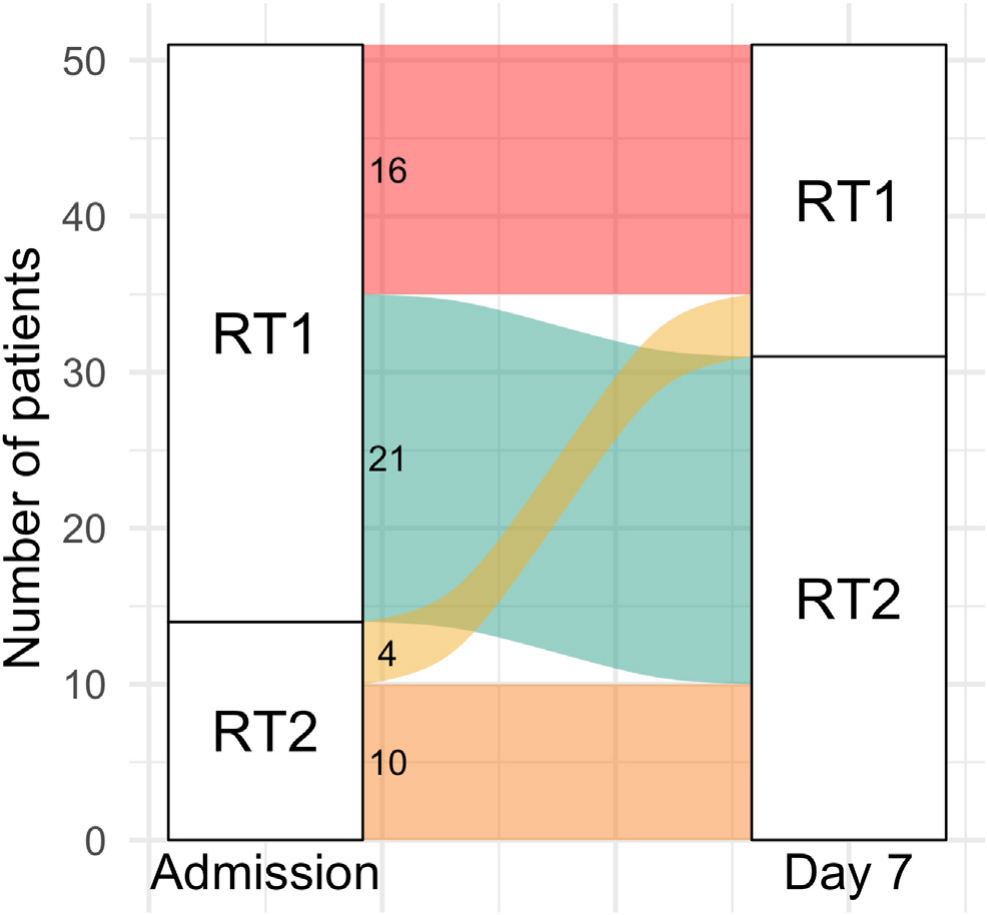
Alluvial plot showing fecal resistotype transitions between time points “Day 0” and “Day 7” Only patients that have their samples collected in two time points are considered. Also see Table S9 for the list of resistotypes assigned to each patient.

Thus, at the time of admission to the hospital (“Day 0”), 61 of 82 patients who were sampled at that time point (74%) had RT1, which is characterized by a higher number of AR genes (81–83 genes on average). That makes 38 of the 51 (74.5%) patients that were sampled both on “Day 0” and “Day 7.” During therapy, 21 of 38 patients with RT1 (55%) underwent a transition to RT2 with a three times fewer number of AR genes (25–30 genes on average) and the macrolide resistance gene *ermB* as a dominant AR gene. 26 of the 51 patients (51%) maintained their initial resistotypes (of those, 17 had RT1 and 9 had RT2 at both time points). Only 4 patients (7.8%) transitioned from RT2 to RT1. An alluvial plot illustrating these resistotype transitions is shown in Figure 11.

Furthermore, a comparison was conducted between the resistotypes observed at the time of hospital admission and their subsequent transitions in relation to disease severity. Of the 61 patients that had RT1 at the time of the admission to the hospital, 30 (49%) got “severe” disease dynamics during the hospital stay, and 31 (51%) got “mild” disease dynamics. Of the 21 patients wtho had RT2 at the time of admission to the hospital, 10 (48%) got “severe” dynamics, and 11 (52%) got “mild” dynamics. Of the 23 patients with the observed resistotype shift within the first week after admission to the hospital, 10 (43%) had “severe” disease dynamics, and 13 (57%) had “mild” disease dynamics. Of the 31 patients that have maintained their initial resistotype 1 week after admission to the hospital, 14 (45%) had “severe” dynamics, and 17 (55%) had “mild” dynamics.

Thus, we could not find a connection between the initial resistotypes or their transitions and the disease dynamics.

The complete data on the resistotypes assigned to each patient at each time point as well as resistotype transitions along with the assigned severity group can be found in Table S9.

#### Antimicrobial resistance gene abundance differences between resistotypes

A comparison of the AR gene compositions of the samples belonging to RT1 and RT2 using DESeq2 revealed 32 genes that were significantly overrepresented in either of the resistotypes. A notable proportion of these genes were beta-lactamases and genes belonging to different efflux systems, including efflux system regulators. Of these, 28 were overrepresented in RT1, and 4 were overrepresented in RT2 (see Figure 13; Table S10).

#### Antimicrobial resistance gene abundance changes over time

The changes in gene abundance that occurred during the course of therapy in each biotope were assessed using the Wilcoxon matched pairs signed rank test. For this analysis, only samples collected on both day 0 and day 7 were used, resulting in a total of 102 paired fecal samples from 51 patients and 168 oropharyngeal samples from 84 patients.

No changes were identified in the oropharyngeal resistome. Given the small variation in the number of AR genes we observed for this biotope, we can suggest that the oropharyngeal resistome is relatively stable and is unlikely to undergo significant changes.

**Figure 12:**
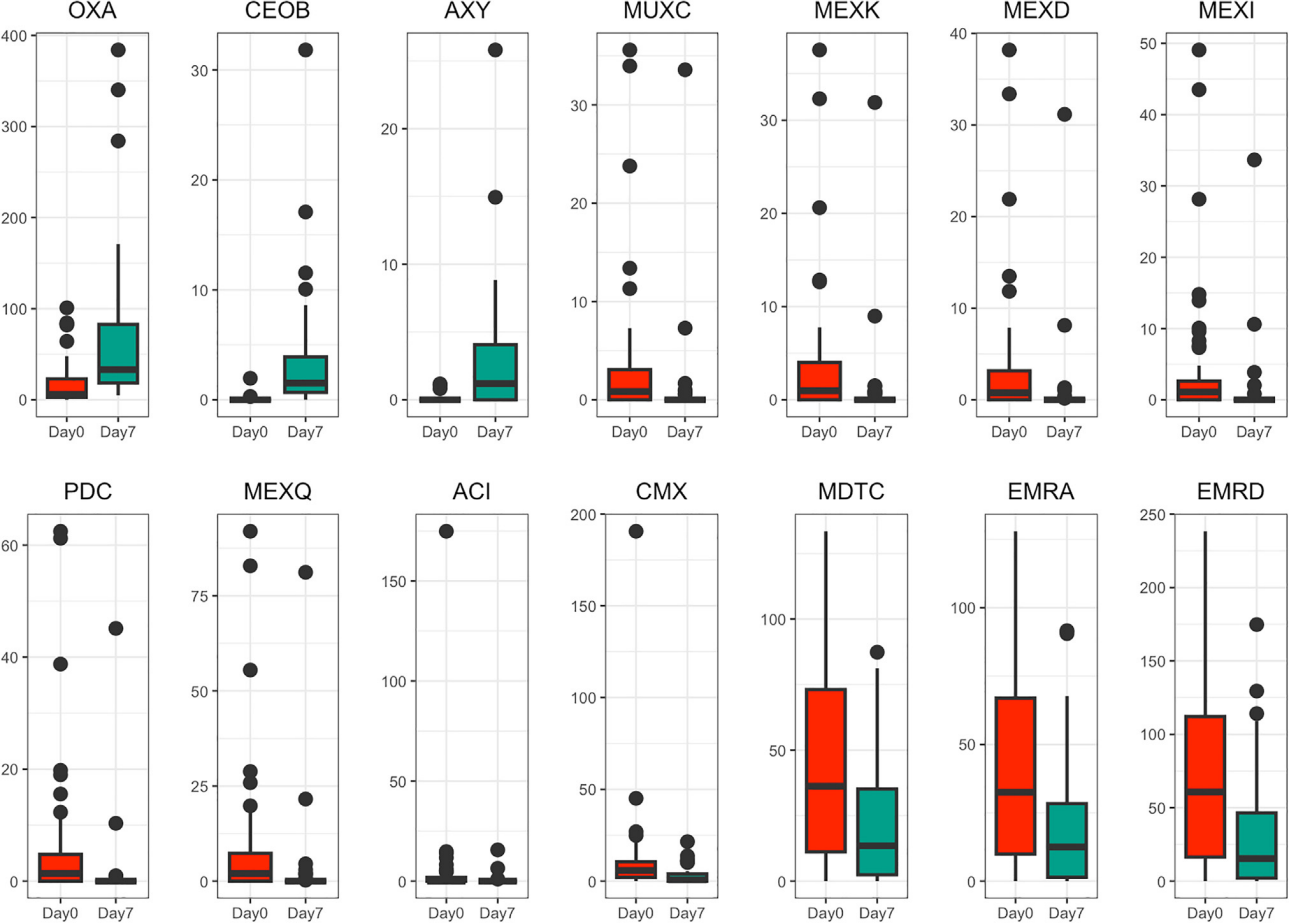
Boxplots of square-root transformed RPKM values for 14 AR genes that were shown to significantly change their abundance between time points in fecal biotopes Also see Table S11 for test details.

In contrast, the fecal resistome appears to be more subject to change. We have identified a total of 14 genes that have significantly changed their abundance during a week of therapy (See Table S11 for test details). These genes include MFS efflux pump genes *emrA*, *emrD*, and *cmx* genes, RND efflux pump genes *mexD*, *mexK*, *mexQ*, *mdtC*, *muxC*, *axy*, *ceoB*, and *mexI* genes, and beta-lactamases *aci*, *oxa*, and *pdc*. One of the genes with the most notable change was the *oxa* gene which considerably increased in abundance on the seventh day. *Oxa* genes analyzed in this study were identified as *oxa*-22 and *oxa*-60, genes previously identified in *Ralstonia*.^10^^,^^11^ Boxplots illustrating abundance changes for each of the genes can be seen in Figure 12.

#### Co-abundant antimicrobial resistance genes

**Figure 13:**
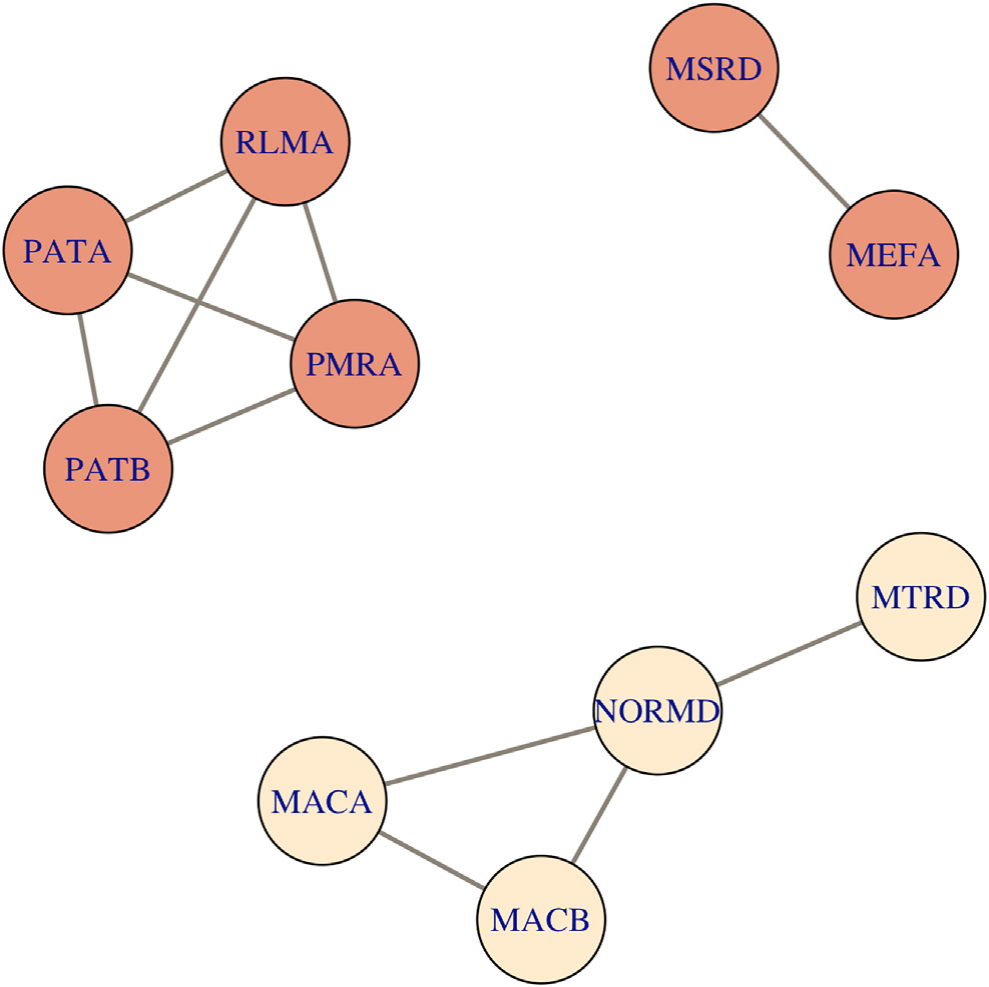
Gene clusters showing high (> 0.9) correlation coefficients based of RPKM values in oropharyngeal samples Pink color represents vertices that are also found in co-abundant gene clusters of fecal samples. Also see Figure S5 for a correlation heatmap and Table S12 for exact correlation coefficients and adjusted *p*-values.

**Figure 14:**
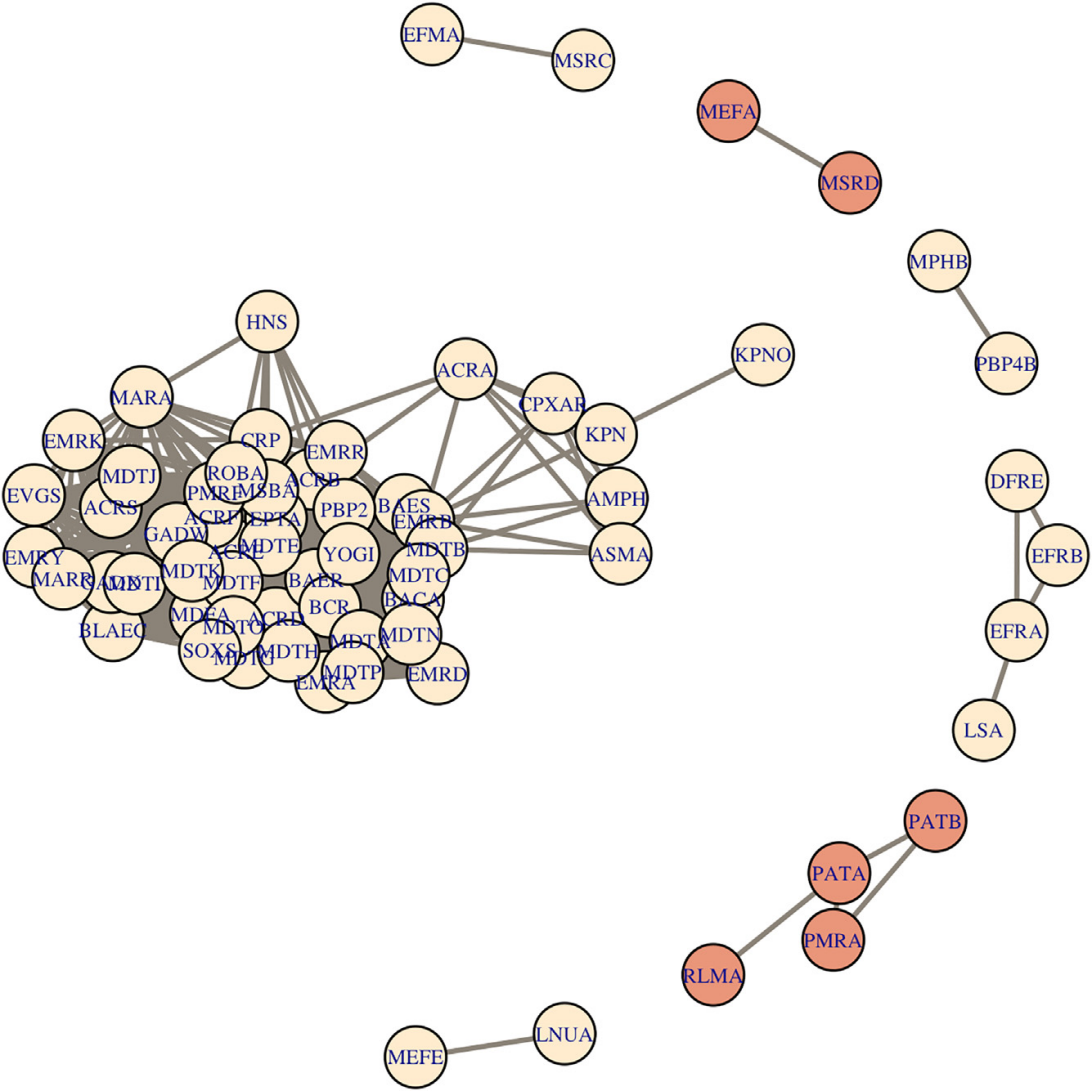
Gene clusters showing high (> 0.9) correlation coefficients based of RPKM values in fecal samples Pink color represents vertices that are also found in co-abundant gene clusters of oropharyngeal samples. Also see Figure S6 for a correlation heatmap, Figures S7 and S8 for co-abundance graphs for each resistotype, and Table S13 for exact correlation coefficients and adjusted *p*-values.

Clusters of co-abundant genes were identified within each biotope and resistotype using Spearman’s rank correlation coefficients. Overall, fecal and oropharyngeal samples showed different patterns of co-abundant gene clusters. There were no large co-abundant gene clusters in oropharyngeal samples (see Figure S5), the largest cluster identified numbered four genes (see Figure 13; Table S12). In fecal samples, there was a large cluster consisting of 52 co-abundant genes (see Figures 14 and S6; Table S13). There were two clusters that were present in both oropharyngeal and fecal samples. The first cluster contained four genes, namely, *patA* and *patB* fluoroquinolone efflux pumps, *rlmA* 23S rRNA methyltransferase, and *pmrA* regulator. The latter is involved in polymyxin B resistance and was also reported to be implicated in multidrug resistance.^12^ The second cluster contained *mefA* and *msrD* ABC efflux pump genes that are involved in macrolide resistance.^13^

Resistotypes 1 and 2 also showed varying patterns of co-abundant gene clusters. We have identified a large gene cluster in RT1 that was partially present in RT2 (see Figures S7 and S8). This cluster numbered 42 and 22 genes in RT1 and RT2, respectively, and contained mostly efflux pump genes and efflux pump regulators. Most of the co-abundant genes present in RT2 were also present in RT1, except for *ermT*–*ermR* genes encoding 23S rRNA methyltransferases, and *efrB*–*dfrE* genes encoding an ABC efflux pump component and a trimethoprim-resistant dihydrofolate reductase, respectively.

### 16S rRNA amplicon sequencing data processing and analysis

#### Enterotypes

**Figure 15:**
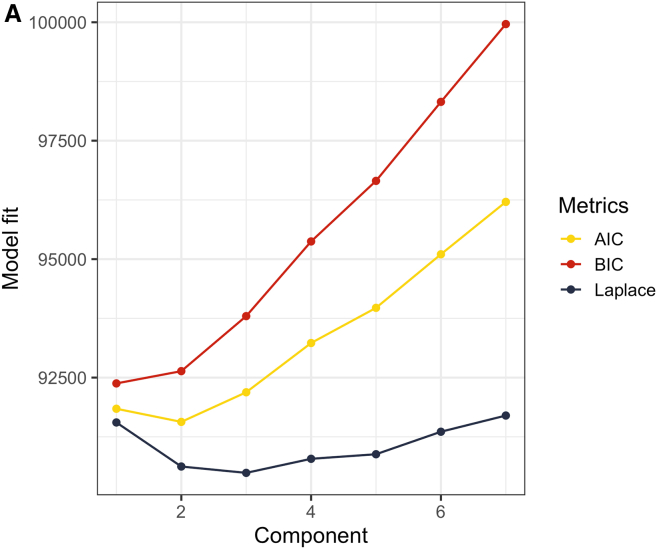
The results of DMM (Dirichlet Multinomial Mixtures) clustering of genus abundance data obtained with 16s rRNA gene amplicon sequencing

We have employed Dirichlet-multinomial mixture modeling to identify potential enterotypes using 16S rRNA gene amplicon sequencing data for fecal samples. The analysis was conducted at the genus level. Laplace approximation of the model fit that showed the lowest value at k = 3 (see Figure 15), suggesting that it is possible to split the samples into 3 enterotypes.

To assess the reproducibility of our findings, we conducted 10 repetitions of the clustering procedure, setting a different random number seed for each repetition. The samples were consistently divided into three enterotypes in seven out of ten instances. These results substantiate the conclusion that the optimal number of clusters is three.

**Figure 16:**
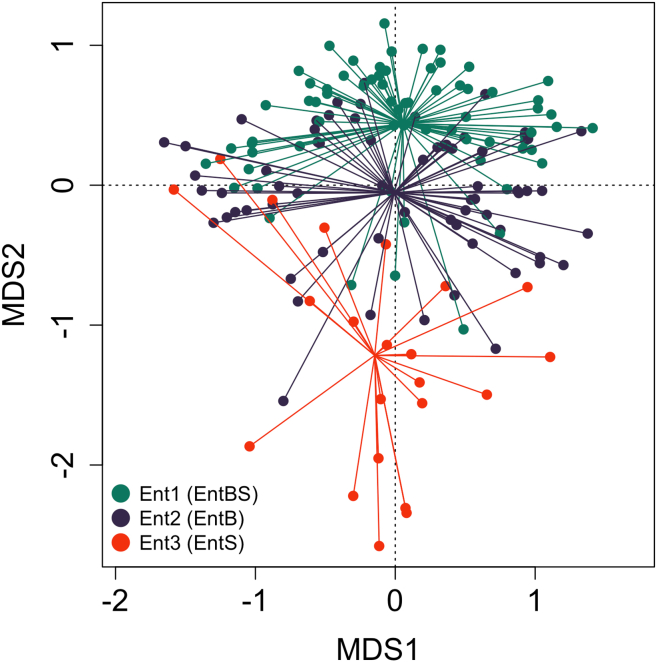
CAP (canonical analysis of principal coordinates) plot representing the Bray-Curtis dissimilarities between samples belonging to the three enterotypes Bray-Curtis distances are calculated based on 16S genera abundance data. Enterotype Ent1 (EntBS) is shown in green, enterotype Ent2 (EntB) is shown in blue, and enterotype Ent3 (EntS) is shown in red. Also see Table S14 for Bray-Curtis distances between the paired samples.

The samples were divided into three enterotypes as follows: Ent1 (*n* = 86 samples, hereinafter “EntBS”), Ent2 (*n* = 53 samples, hereinafter “EntB”), and Ent3 (*n* = 18 samples, hereinafter “EntS”). The dissimilarity of the three enterotypes is shown on a PCoA plot in Figure 16.

**Figure 17:**
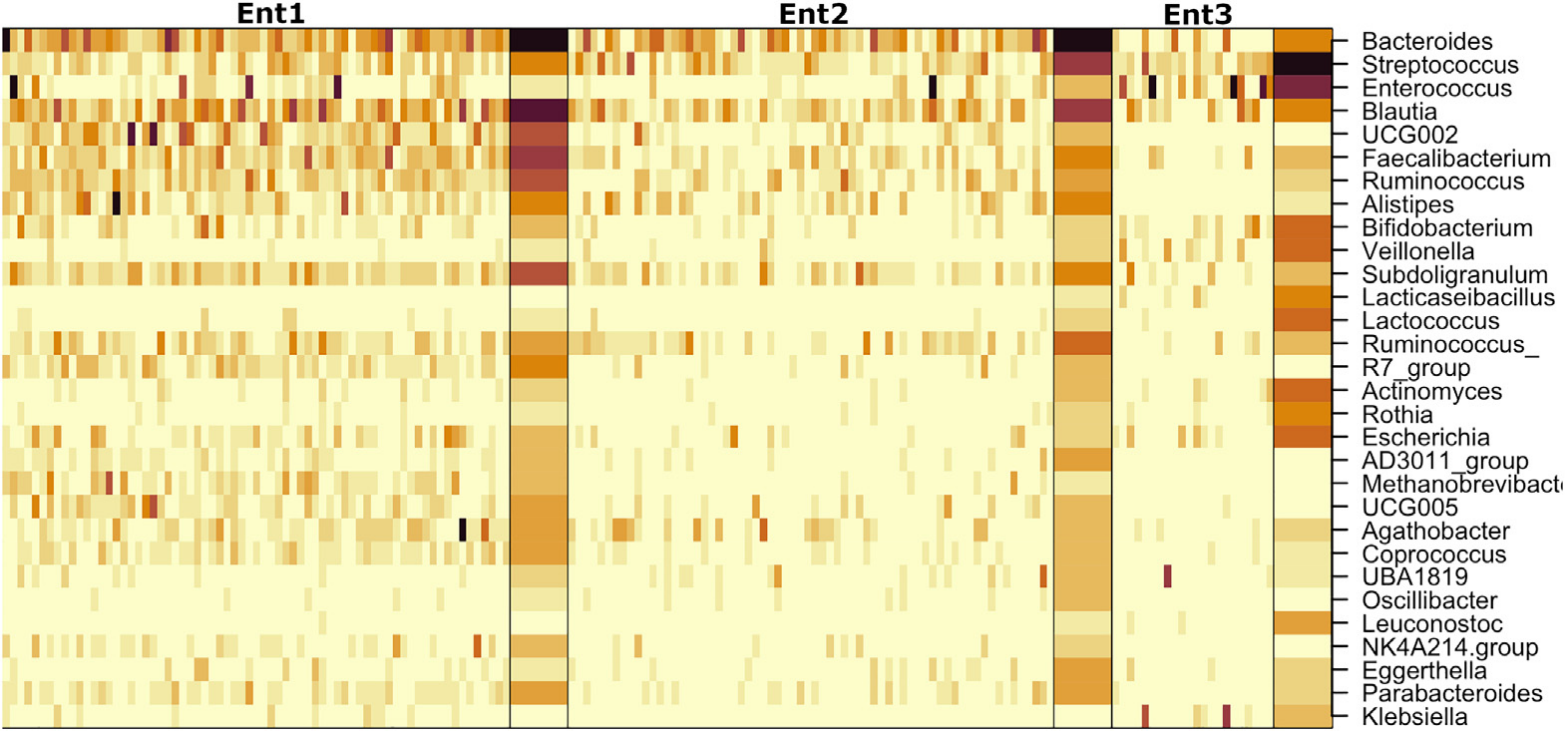
A heatmap shows the most abundant AR genes in fecal samples in the three identified fecal enterotypes The heatmap is based on square-root transformed RPKM values. Columns correspond to samples, and samples are grouped by Dirichlet component, with means summarized as a separate wide column to the right of each group. Darker colors correspond to higher RPKM values. Also see Figure S9 for enterotype drivers.

EntB was characterized by high abundances of *Bacteroides*, *Ruminococcus*, *Blautia*, and *Faecalibacterium*. EntBS was characterized by high abundances of *Bacteroides*, *Ruminococcus*, *Blautia*, and *Streptococcus*. EntS was notably different from the previous two and had *Enterococcus* and *Streptococcus* as the most abundant genera (see Figure 17 for a heatmap; Figure S9 for the top drivers).

**Figure 18:**
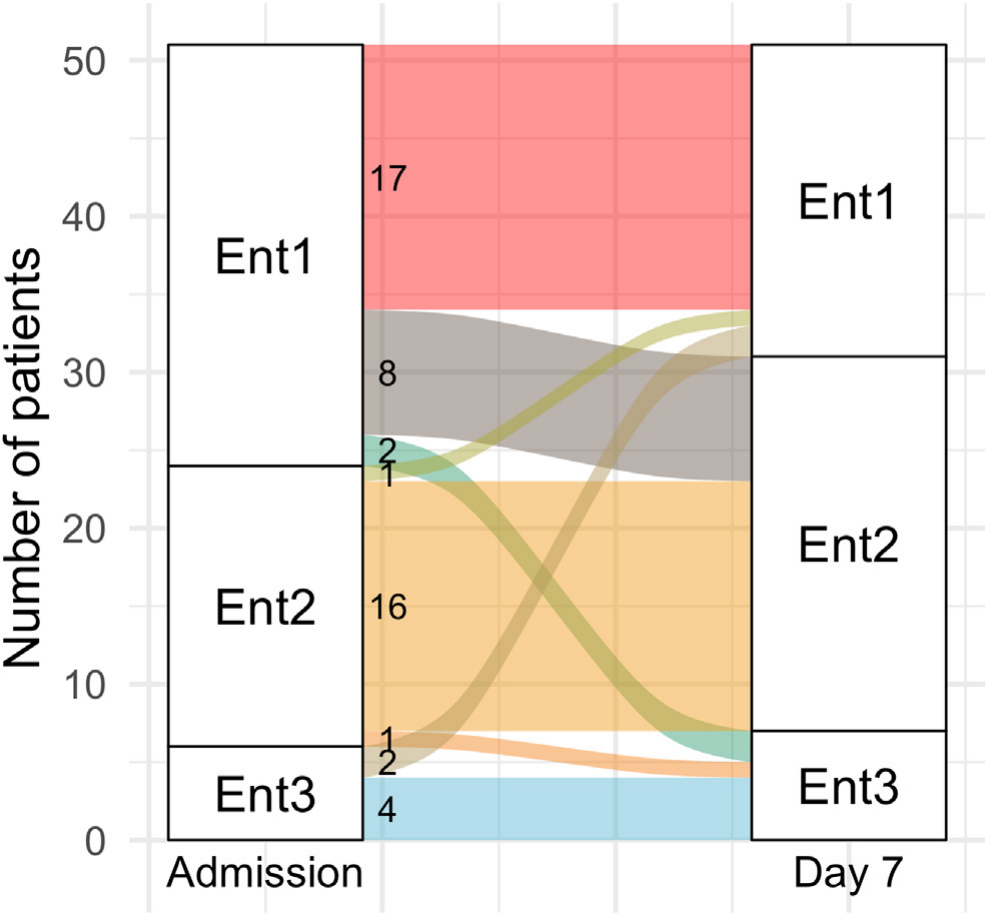
Alluvial plot shows fecal enterotype transitions within a week of therapy Only patients that have their samples collected in two time points are considered. Also, see Table S9 for the list of enterotypes assigned to each patient.

We compared enterotypes assigned to the same patients at different time points and found that the majority of patients (89%) who had enterotype 2 (EntB) upon admission to the hospital (“Day 0”) maintained it one week later (“Day 7”). Meanwhile, approximately 30% of patients with enterotype 1 (EntBS) upon admission changed to EntBS a week later (see Figure 18 for an alluvial plot). However, the Chi-square test did not reveal any significant association between the observed enterotypes and the sampling day (*p*-value = 0.8303).

Furthermore, no connection could be established between the observed enterotypes and disease severity.

Detailed data on the enterotypes assigned to each patient at each time point as well as enterotype transitions along with the assigned severity group can be found in Table S9.

#### Bacterial diversity

**Figure 19:**
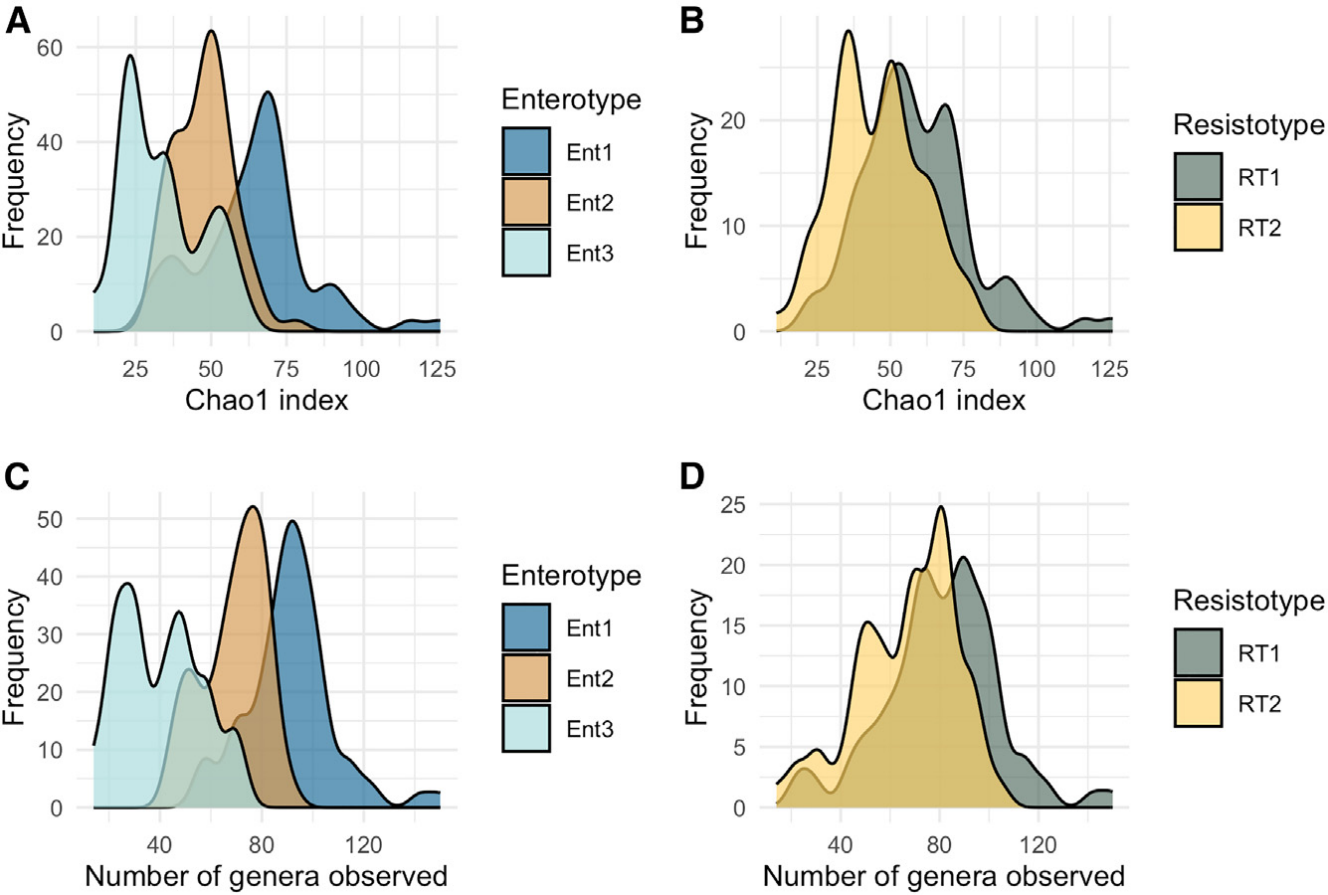
Bacterial diversity metrics estimated for the fecal samples (A) Species diversity estimated as Chao1 index, samples grouped by enterotypes. (B) Species diversity estimated as Chao1 index, samples grouped by resistotypes. (C) Number of genera observed, samples grouped by entrotypes. (D) Number of genera observed, samples grouped by resistotypes It can be seen that resistotype RT1 is associated with higher bacterial diversity, meanwhile resistotype RT2 is associated with lower bacterial diversity Also see Figure S10 for bacterial diversity metrics estimated for the oropharyngeal samples.

Bacterial diversity estimated for oropharyngeal and fecal data using Chao1 indices and the number of observed genera is illustrated in Figures 19 and S10. Using Kruskall-Wallis rank-sum test, we found that diversity indices significantly differ between the three enterotypes. It can be seen in Figure 19, that enterotype 1 (EntBS) is associated with higher bacterial diversity, meanwhile enterotype 3 (EntS) is associated with lower bacterial diversity.

#### Taxa abundance changes over time

We assessed changes in bacterial species and genera abundances in patients during the first week of therapy using the Wilcoxon matched-pairs signed-rank test. For this analysis, we only used paired samples that were collected at the two time points: 102 paired fecal samples collected from 51 patients and 168 oropharyngeal samples collected from 84 patients.

No changes were identified in the oropharyngeal samples at the genus or species levels. As for fecal samples, we have identified four species and four genera that had significant differences in their abundance between the two time points (see Table 4). The most notable change was the decrease in the abundance of *Blautia* and *Blautia obeum*, specifically, at the second time point. The observed increase in abundance of fecal *Ralstonia* aligns with the increase in abundance of fecal *oxa*-60 and *oxa*-22 carbapenemase genes (see “antimicrobial resistance gene abundance changes over time” subsection), although *Ralstonia* was not detectable in the first time point in fecal samples and had quite low abundance at the second time point, unlike *oxa* genes.

**Table 4:** Bacterial taxa that differed significantly in abundance between “Day 0” and “Day 7” time points in fecal samples

Genus	Adjusted *p*-value	Median in point 1	Median in point 2
*Blautia*	0.001325102019	3191	441.5
UCG-002^a^	0.003764494301	246.5	1022.5
*Ralstonia*	0.000005335958	0	30
*Senegalimassilia*	0.001372494724	40	0.5
Species	Adjusted *p*-value	Median in point 1	Median in point 2
*Blautia obeum*	0.000956556383	1064	172
*Bifidobacterium adolescentis*	0.006685909590	5	0
*Dorea formicigenerans*	0.007151366839	103.5	10.5
*Ralstonia insidiosa*	0.000005561421	0	30

The differences were identified using the Wilcoxon matched-pairs signed-rank test. Assigned taxonomy is provided according to the SILVA database (v138).

a uncultured bacteria currently assigned to *Oscillospiraceae* family.

#### Sample dissimilarity

To determine if the samples taken from the same patient had higher similarity to each other than to any other samples, we calculated Bray-Curtis distances based on genus abundance data. We have assigned the “closest” sample with the minimal Bray-Curtis distance for each of the “paired” fecal and oropharyngeal samples. Eleven out of the 98 (11.22%) fecal samples and two out of the 168 (1.19%) oropharyngeal samples showed the highest similarity to their “paired” samples (see Table S14).

### Connecting antimicrobial resistance gene data with taxonomy data

#### Abundance correlations

We attempted to identify potential correlations between AR gene abundance data (RPKM values) and bacterial taxa abundance data obtained via 16S rRNA amplicon sequencing. However, we were not able to identify any significant (p ⟨< 0.05) correlations in oropharyngeal or fecal samples.

#### Bacterial composition differences between resistotypes

**Figure 20:**
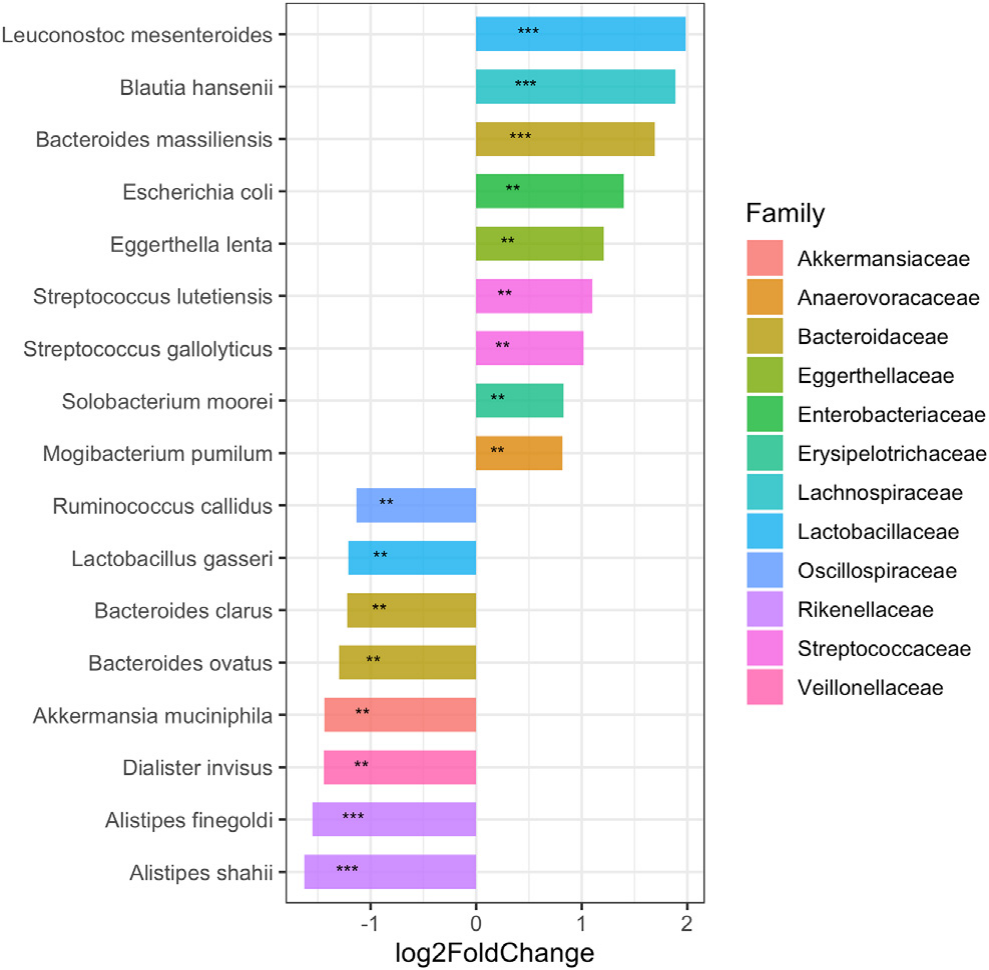
Barplots illustrating log2 fold change of species counts between resistotypes 1 and 2 calculated during DESeq2 analysis Bars oriented right (positive log2FC) correspond to species overrepresented in RT2, bars oriented left (negative log2FC) correspond to species overrepresented in RT1. Species with *p*-values ⟨< 0.05 are marked with “∗∗,” and species with *p*-values ⟨< 0.01 are marked with “∗∗∗.” For details, see Table S16. For genus and ASV levels, see Figures S13 and S14 and Table S15.

We assessed differences in bacterial composition between the two resistotypes using DESeq2. Eight ASVs, three species, and 12 genera were identified as being overrepresented in RT2 in comparison to RT1. At the same time, we have identified 12 ASVs, two species, and 16 genera that were overrepresented in RT1 as compared to RT2 (see Figure 20 for the species level; Figures S13 and S14 for the genus and ASV levels, correspondingly). Bacterial taxa that were found to be associated with specific resistotypes are listed in Tables S15 and S16.

#### Bacterial diversity

Bacterial diversity was estimated for each resistotype as Chao1 indices and the number of the observed genera is illustrated in Figures 19B and 19D. Using the Mann-Whitney U test, we found that diversity indices differ significantly between the two resistotypes. The measures of bacterial diversity estimated for fecal samples as well as the results of the statistical tests.

#### Self-similarity and enterotypes

We have checked whether the number of AR genes and AR gene variants at the time of admission to the hospital correlated with Bray-Curtis distances between the first and the second time points, as calculated based on sample genus composition (see Figures S11 and S12). We did not find any significant correlation.

We also compared the numbers of AR genes at the time of admission to the hospital between the patients who did and did not change their enterotype. We did not find a significant difference between the two groups.

**Figure 21:**
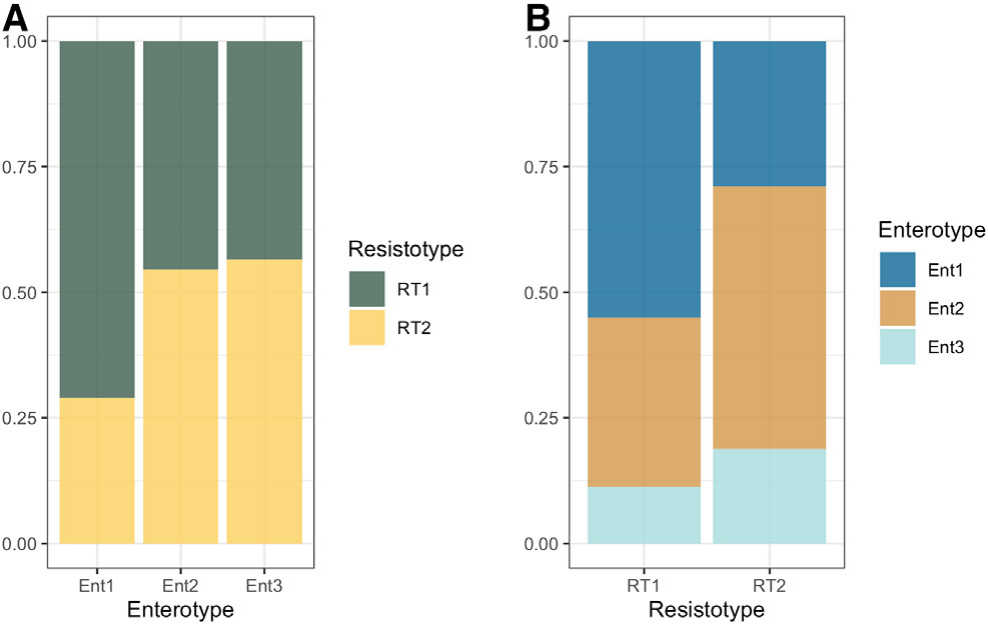
Barplots show how the three identified enterotypes correspond to resistotypes Y axis shows (A) the ratio of the samples assigned to certain resistotypes within each enterotype (B) the ratio of samples assigned to certain enterotypes within each resistotype.

Interestingly, we found a connection between resistotypes and enterotypes, as shown by the Chi-squared test (*p*-value = 0.003747). As illustrated in Figure 21, the majority of samples (72%) assigned to enterotype 1 (EntBS) also belong to resistotype RT1. This aligns with the preceding observation that both resistotype RT1 and enterotype 1 (EntBS) are associated with higher bacterial diversity.

### Antibiotic intake impact

To investigate the potential influence of antibiotic administration on the observed alterations in fecal resistome, we stratified the patients into two cohorts based on the presence or absence of antibiotic therapy: the antibiotic group (*n* = 22) and the non-antibiotic group (*n* = 28).

We applied Wilcoxon matched-pairs signed-rank tests to detect antibiotic resistance genes that showed significant changes in abundance between the two sampling time points (“Day 0” and “Day 7”) within each group. In the antibiotic group, only one gene (*ceoB*) displayed change with adjusted *p*-value of 0.041. Within the non-antibiotic group, two genes (the same *ceoB* gene, and *oxa* beta-lactamase gene) showed possible changes with adjusted *p*-values of 0.012 and 0.023.

Additionally, we conducted Mann-Whitney U tests based on Bray-Curtis distances measured between paired samples using both 16S genera abundance data and resistome AR gene abundance data. We compared the means of these distance metrics between the two groups. Our results did not allow us to reject the null hypothesis for either the resistome data (*p*-value = 0.9766) or the genus abundance data (*p*-value = 0.3205).

**Figure 22:**
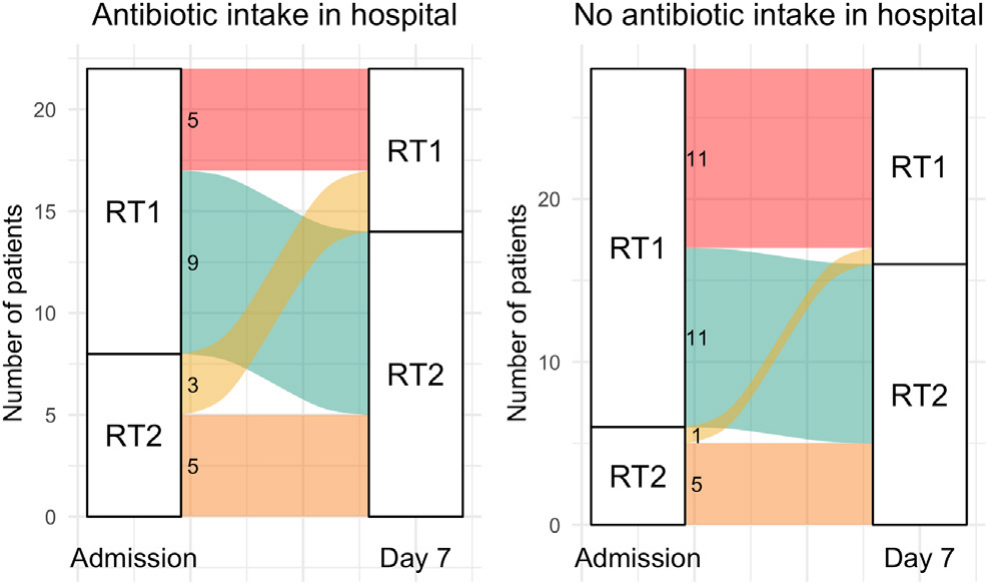
Alluvial plot show fecal resistotype transitions within a week of therapy in antibiotics and no-antibiotics groups Also see Table S9 for the list of resistotypes and antibiotics intake groups assigned to each patient.

This suggests that antibiotic intake does not appear to affect the similarity of paired samples within patients. Finally, a Chi-square test was conducted to compare the frequencies of resistome shifts in each group (see Figure 22 for alluvial plots). We could not reject the null hypothesis (*p*-value = 0.4355), suggesting that the patients might exhibit similar patterns of resistome changes during COVID-19 therapy regardless of whether or not they were taking antibiotics.

## Discussion

In our research, we assessed the resistomes and microbiomes of the gut and oropharynx in patients diagnosed with COVID-19. While the majority of studies examining the total resistomes of microbial communities within the human body rely on shotgun metagenomics sequencing data,^9^^,^^14^^,^^15^ this study employs a more sensitive approach to specifically capture AR gene sequences. We have designed a targeted sequencing panel that allows for the analysis of 4,937 selected antibiotic resistance determinants simultaneously, and we supplemented the AR gene targeted sequencing data with 16S rRNA gene amplicon sequencing data. This targeted and sensitive approach allowed us to specifically assess the two sets of the sequences of our interest.

Using this method, we investigated the changes in the fecal and oropharyngeal resistomes of patients diagnosed with COVID-19 over the course of therapy and explored their connections with the taxonomic composition of the samples. To our knowledge, this is one of the first descriptions of the oropharyngeal resistome, and the first study to assess oropharyngeal resistome dynamics over time. We identified 7 AR genes that are most widespread in the oropharynx as well as 7 genes that are most widespread in the gut. Three of those genes, namely, *ermB*, *msrD*, and *mefA*, were present in more than 80% of both oropharyngeal and fecal samples.

We have identified three “core” genes that were present in most of the oropharyngeal and fecal samples at the same time, namely, 23S rRNA methyltransferase *ermB*, and efflux system genes *msrD* and *mefA*. These genes belong to multiple hosts, but in our data, all the three genes have sequence variants that are found in streptococci. Streptococci, such as *Streptococcus salivarius*, are common in airways,^16^ but are also gut commensals.^17^ These bacteria have been shown to increase their abundance in gut microbiomes in patients receiving proton pump inhibitors (PPIs) such as omeprazole.^18^ PPIs lower the barrier function of the stomach, leading to the increased survival of the oropharyngeal bacteria in the intestine.^19^ Since the patients from the current study received omeprazole as a part of their therapy scheme, and streptococci were highly abundant in both oropharyngeal and fecal samples, it is possible to suggest that some sequence variants of the fecal *ermB*, *msrD* and *mefA* might have originated from the oropharyngeal streptococci.

At the same time, we have found four “core” AR genes that were only widespread in the oropharynx, namely, class A beta-lactamase *cfx*, class 2 ABC transporter *lsaC*, *patA* ABC efflux pump gene, and 23S rRNA methyltransferase *ermX*. The *cfx* gene is found in several bacterial genera, mainly in *Bacteroides* and *Prevotella*, which were both identified in oropharyngeal samples.^20^^,^^21^ It is considered mobilizable due to its association with the conjugative transposon Tn*4555*^21^ and possibly with other mobile elements.^22^ This gene was found in 97% of the oropharyngeal samples, and we can assume that its wide dissemination in the oropharynx might be a result of the activity of the mobile elements. The *lsaC* gene, which is associated with lincosamide and streptogramin A resistance, has only been reported in group B streptococci, carried by a family of integrative and mobilizable elements (IMEs).^23^ The *patA* is part of a streptococcal ABC efflux system that is associated with fluoroquinolone resistance.^24^ The *ermX* gene determines resistance to macrolides in *Corynebacterium*, which is commonly found in the airways.^25^ *ErmX* and *cfx* genes (but not *patA* and *lsaC* genes) were previously reported as a part of the “core” resistome of the airways.^26^ Thus, the “core” resistome of the oropharynx can most likely be explained by the high prevalence of certain commensal bacteria in the airways.

Analyzing the oropharyngeal resistome, we have noted its relatively low dispersion. We were not able to identify any resistotypes in the oropharyngeal samples, nor were we able to identify significant changes in certain AR gene abundances through therapy. However, the oropharyngeal resistome was not stable over time, as the samples taken from the same individuals at different time points were rarely similar, with an overall self-similarity ratio of 8%. By comparison, the oropharyngeal microbiome self-similarity ratio was even lower (1%). These results suggest that the oropharyngeal resistomes and microbiomes are changing systems, especially under conditions of high drug load. Given that they are characterized by relatively low variance, as described above, we can suggest that the changes in the oropharyngeal resistomes and microbiomes of the same individuals occur within a narrow range of possible compositions.

Interestingly, the fecal resistome showed a relatively higher self-similarity ratio than that of the oropharyngeal resistome (18% vs. 8%) and much higher variance at the same time. The self-similarity ratio of the fecal microbiome was 11%. We also found that the changes that the fecal microbiomes underwent during one week of therapy were not likely to be influenced by the initial number of AR genes in the microbial community. Overall, both resistome and microbiome samples from both biotopes showed low self-similarity. This might be partly explained by a custom selection of reference AR genes, where we excluded the most widespread sequences, thus, we were more likely to detect the variation.

One of the findings of our study is the identification of the two distinct resistotypes in the fecal samples that were apparently different in their AR gene number and composition. In our study, one of the two resistotypes had three times fewer AR genes than the other and was strongly dominated by a macrolide resistance 23S rRNA methyltransferase *ermB* gene. This drastic difference in the diversity of AR genes between resistotypes could be partly, but not entirely explained by the difference in bacterial alpha diversity of the samples. Having compared the AR gene compositions of the two sets of samples belonging to resistotypes 1 and 2, we found that RT2 lacks many AR genes that are present in RT1, but at the same time, it shows a relatively higher abundance of *lsaC*, a streptococcal MLS resistance gene that is a part of the “core” resistome of the oropharynx, as we showed above (see Figure 13). Three other genes overrepresented in RT2 were *mdtJ* and *mvrC* small multidrug resistance (SMR) efflux pump genes, and *gadX* gene encoding a transcriptional regulator for RND efflux systems. All the three genes were reported in *Escherichia*/*Shigella*.^27^^,^^28^^,^^29^

Having analyzed the co-abundance AR gene clusters of resistotypes 1 and 2, we noted a large cluster of co-abundant efflux pump genes in RT1 that was only partly present in RT2 (see Figures S12 and S13). Two of these genes were *gadX* and *mdtJ*, which were overrepresented in RT2, while none of these genes were overrepresented in RT1. Interestingly, core resistome genes *mefA* and *msrD* genes were highly correlated in their abundance in resistotype RT1, but not in RT2. These two genes are generally found in one operon associated with macrolide resistancee.^13^ This might indicate that at least one of the two genes is possibly present in a different genomic context in RT2.

We have also compared the taxonomic composition of the samples belonging to the two resistotypes. Of the 8 species overrepresented in RT2, we have noted *Leuconostoc mesenteroides*, *Blautia hansenii*, *Escherichia coli*, and *Streptococcus gallolyticus* (see Figure 20). The presence of *ermB* gene associated with mobile genetic elements such as plasmids and Tn*916*-like conjugative transposons was shown for all of the four species.^30^^,^^31^^,^^32^^,^^33^ Of those, *L. mesenteroides* was able to transfer *ermB*-containing conjugative elements to *Enterococcus faecalis* both in laboratory conditions and on a food matrix.^30^ The increased abundance of *E.coli* in resistotype RT2 may be linked to the increased abundances of *mvrC*, *mdtJ*, and *gadX* efflux system genes in the same resistotype that was discussed above. Some strains of the genera *Weissella* and *Eggerthella* that were overrepresented in RT2 (see Figure S13 were also shown to contain *ermB* genes.^34^^,^^35^ Given these results, we can suggest that *ermB* gene found in resistotype RT2 might possibly exist in a different set of genomic contexts than in RT1.

Our findings align with one of the previous studies. In a recent article, Lee et al. have identified two resistotypes as a part of the population-level bioinformatics study assessing the impact of per capita antibiotic usage rate on human gut resistomes across ten countries.^36^ Although we cannot compare our results directly due to the different methods used and the different sets of the AR genes analyzed, we find it worth noting that the two resistotypes identified differed in overall AR gene abundance. Interestingly, the researchers found an association between enterotypes and resistotypes, although it was weak. Nevertheless, the researchers suggested that resistotypes are independent of the enterotypes.

In an earlier study, other researchers have identified six resistotypes in human gut metagenomes based on MEGAHit shotgun metagenomic samples and linked them to the six identified enterotypes.^37^

In our data, we were able to identify three enterotypes, with two of them being dominated by *Bacteroides*, *Blautia* and *Ruminococcus* and the third being dominated by enterococci and streptococci. *Bacteroides*-dominated enterotypes were reproducibly reported in earlier microbiome studies.^38^^,^^39^ However, *Streptococcus*- or *Enterococcus*-dominated enterotypes have never been reported in previous research. Due to the small number of samples in entS (*n* = 18) and its heterogeneity (see Figure 16) as well as the nature of our samples, we can assume that it might not be a true enterotype but an abnormal pattern associated with low bacterial diversity and decreased barrier function of the digestive system, resulting in the enrichment of the oropharyngeal streptococci. We did not identify any *Prevotella*-dominated enterotypes in our data, and the overall abundance of *Prevotella* was very low.

In our research, we have also found a connection between resistotypes and enterotypes, as shown by the Chi-squared test. Figure 21 shows a visible link between one of the identified enterotypes (EntBS) and a certain resistotype, namely, RT1, with most of the EntBS samples (72%) also belonging to this resistotype. However, since we could not identify any other noticeable linkage between resistotypes and enterotypes, we can speculate that resistotypes and enterotypes only have a limited connection and do not correlate strongly.

Another key finding of our study is the observed resistotype shifts that the patients underwent through drug therapy. This is one of the first studies assessing resistotype dynamics over time.

We discovered that approximately half of the patients changed their resistotype within the first week of therapy in a hospital setting. Most of these patients shifted from RT1 to RT2, with a lower number of AR genes and a marked prevalence of *ermB* gene. In other words, their gut microbiomes had lost about two-thirds of the AR genes present at the time of the admission to the hospital. This might be explained by the depletion of the hosts of these genes that occurred during the course of the disease. Given the multihost nature of the *ermB* gene, we can assume that the resistotype shift from RT1 to RT2 may as well be accompanied by the change in the host range of the *ermB* gene.

Similarly, several patients have undergone enterotype shifts (see Figure 18), transiting from enterotype 1 (EntBS) to enterotype 2 (EntB). However, we could not find any significant association between the observed enterotypes composition and the sampling day. These results suggest that the fecal resistome has undergone more considerable changes than the fecal microbiome.

When comparing the resistome and microbiome data with the patients’ metadata, we were unable to find a connection between the observed resistotype and enterotype changes and the severity of disease course, possibly due to the limitations of our data, as COVID-19 often comes with comorbidities that might as well affect the gut microbiome. Studies involving different patient cohorts may provide insight into the potential link between COVID-19 and the fecal resistome.

Interestingly, we could not establish a connection between the observed resistotype shifts and antibiotic intake during therapy (see Figure 22). This suggests a different driver for the resistome changes reported in this study, possibly connected to an overall drug load during hospital therapy or particular non-antibiotic drugs used in therapy regimens. One of the possible drivers contributing to the resistome changes might be proton pump inhibitors, which could promote the transition of the drug-resistant oropharyngeal microbes to the gut.^18^ This hypothesis is consistent with the fact that specific streptococcal sequence variants of several AR genes were found in both oropharyngeal and fecal samples. However, other factors that could influence resistome changes during hospital therapy should be considered in future studies.

In conclusion, given the distinct resistotypes identified and the significant variance observed, we can infer that fecal resistomes are dynamic systems, but the changes observed in our study apparently occur within certain ranges of possible combinations, or states. While these changes are in all likelihood influenced by the taxonomical composition of the samples, resistotypes only have a limited connection with enterotypes, possibly due to the fact that many AR genes are present on mobile genetic elements that can be shared between different bacterial hosts. So, we can suggest that resistome is a part of a microbiome that can have its own properties that are only partly dependent on the taxonomic composition of the microbiome. We can also assume that microbiome and resistome can change differently under certain circumstances. We have observed resistome changes that we could not fully explain by the taxonomic composition of the samples. The underlying mechanisms of such changes and the triggers that provoke it have yet to be determined.

### Limitations of the study

Our study claims to characterize the resistomes of the oropharyngeal and fecal microbiomes. However, we only focused on 4,937 antibiotics resistance determinant sequences that were selected from the MEGARes 3.0 database. We did not assess sequences linked to tetracycline and aminoglycoside resistance because of their wide dissemination in fecal samples. We also realize that antibiotics resistance gene databases such as MEGARes might be biased toward well-studied pathogenic bacteria and model organisms such as *E. coli* and contain very few AR gene sequences belonging to gut commensals. Some studies suggest that AR genes belonging to gut commensals show low identity to known AR genes found in AR gene databases, so, most likely, they were not captured with our AR gene panel. So, the set of AR genes present in our analysis is far from being exhaustive. We believe that future researchers will address this issue and include a comprehensive set of AR gene sequences belonging to commensal bacteria in AR gene databases. We also did not consider such resistance mechanisms as antibiotic’s target gene point mutations. Another limitation is that we only track resistome changes over a short period of time (1–2 weeks). Although we were able to detect resistotype shifts, we cannot tell if these shifts are permanent or temporary just based on the given data.

## Resource availability

### Lead contact

Requests for further information and resources should be directed to and will be fulfilled by the lead contact, Elizaveta V. Starikova (hed.robin@gmail.com).

### Materials availability

This study did not generate new materials.

### Data and code availability

• Sequencing reads obtained for resistome samples are deposited into NCBI as BioProject PRJNA1005621 and are publicly available by the following link: https://www.ncbi.nlm.nih.gov/bioproject/PRJNA1005621. Sequencing reads for 16s rRNA gene amplicons were deposited into NCBI as BioProject PRJNA989180 and are publicly available by the following link: https://www.ncbi.nlm.nih.gov/bioproject/PRJNA989180/. The reference set of the AR gene sequences used for targeted gene panel design are publicly available at figshare repository by the following link: https://doi.org/10.6084/m9.figshare.23744580.v1. All other data reported in this article will be shared by the lead contact upon request.
• Our Snakemake workflow for creating resistome profiles for each sample can be found at the GitHub repository by the following link: https://github.com/bobeobibo/AR-panel-snake. R code used for the subsequent analysis of resistome profiles is available as an R markdown document by the following link: https://doi.org/10.6084/m9.figshare.25250899.v1.
• Any additional information required to reanalyze the data reported in this article is available from the lead contact upon request.

## Acknowledgments

This work was supported by State Assignments No. 122040100011-0 and No. 122030900064-9. The major part of this work was performed using the core facilities of the Lopukhin FRCC PCM “Genomics, proteomics, metabolomics” (http://rcpcm.org/?p=2806).

## Author contributions

E.V.S. analyzed the resistome and 16S rRNA gene amplicon datasets and wrote the article. Y.S.G. and D.E.F. analyzed the 16S rRNA gene amplicon datasets. E.V.K. and A.S.S. performed sequencing of the resistome samples. O.V.S., P.Y.Z., K.M.K., V.A.V., M.D.M., and D.I.B. performed DNA extraction and library preparation for all samples and sequencing of the 16S rRNA gene amplicon samples. E.I.O. performed the targeted gene panel design. I.E.K. and A.I.M. contributed to the resistome dataset analysis. A.V.P. managed sample collection and storage. O.O.Y., N.I.K., O.V.L., D.N.A., F.S.S., A.K.F., M.K.D., N.G.A., A.V.Z., S.V.T., V.V.E., and P.A.B. collected samples and metadata. I.V.M. contributed to the study design. V.M.G. contributed resources. E.N.I. designed and supervised the study. All the authors have read and approved the final article.

## Declaration of interests

The authors declare no competing interests.

## STAR★Methods

### Key resources table

**Table:** 

REAGENT or RESOURCE	SOURCE	IDENTIFIER
**Biological samples**		

Stool samples of 100 COVID-19 patients	Clinical Medical Center “Kuskovo” of the Moscow State University of Medicine and Dentistry (MSUMD)	NA

**Deposited data**		

Resistome raw sequence data	Current study	NCBI BioProject: PRJNA1005621
16S rRNA gene amplicon sequencing data	Current study	NCBI BioProject: PRJNA989180
MEGARes database	https://www.meglab.org/	version 2.0
SILVA database	https://www.arb-silva.de/	version 138

**Software and algorithms**		

Trimmomatic	https://github.com/usadellab/Trimmomatic	version 0.39–2
bowtie2	https://github.com/BenLangmead/bowtie2	version 2.4.2
R	https://www.rproject.org/	version 4.2.2
Snakemake workflow for creating resistome profiles used in this study	Current study	https://github.com/bobeobibo/AR-panel-snake
samtools	http://www.htslib.org	version 1.6
DirichletMultinomial R package	https://github.com/mtmorgan/DirichletMultinomial	version 1.40.0
Custom R code and used in this study	Current study	https://figshare.com/articles/software/Targeted_AR_gene_panel_sequencing_data_analysis_R_markdown_/25250899
vegan R package	https://github.com/vegandevs/vegan	version 2.6–4
DESeq2 R package	https://bioconductor.org/packages/release/bioc/html/DESeq2.html	version 1.38.3

### Experimental model and study participant details

#### Patients details

The study was conducted as part of a project evaluating the microbiomes of COVID-19 patients^40^ The study included one hundred COVID-19 patients with a confirmed SARS-CoV-2 infection that were admitted to the Clinical Medical Center “Kuskovo” of the Moscow State University of Medicine and Dentistry (MSUMD) from April to June 2021. The study population included patients aged 18 or older. The cohort consisted of 49 males and 51 females, with an age range between 25 and 88 years. All patients enrolled in the study gave informed consent to participate. The standard treatment regimens included glucocorticoids, mucolytics, anticoagulants, non-steroidal anti-inflammatory drugs and proton pump inhibitors. The patients were divided into two groups according to the severity of their disease course: “severe” and “mild”. “Mild” patients had stable disease dynamics, while “severe” patients had episodes of negative dynamics after admission to the hospital, such as.

(1) the progression of pneumonia (as detected with computed tomography)
(2) development of the disease complications
(3) transfer to the intensive care unit
(4) clinical conditions requiring enhances pharmacotherapy and interventional treatment, such as additional oxygen insufflation and mechanical ventilation
(5) fatal outcome

The severity groups assigned to each patient can be seen in Table S9.

The study was approved by the Independent Interdisciplinary Ethics Committee on Ethical Review for Clinical Studies (http://ethicuni.ru/about.php?l=0, protocol No. 01–21 from 28.01.2021). All the patients who participated in this study provided informed consent to participate. All the experiments were performed in accordance with the relevant guidelines and regulations.

### Method details

The overall experiment scheme can be found in Figure 1.

#### Sample collection

The samples from 100 COVID-19 patients with a confirmed SARS-CoV-2 infection were collected at the Clinical Medical Center “Kuskovo” of the Moscow State University of Medicine and Dentistry (MSUMD) between April and June 2021.

Oropharyngeal samples were collected with cotton swabs from the affected areas of the oropharynx, including the tonsils, the arms of the soft palate, the uvula and the posterior pharyngeal wall. Fecal samples were collected in sterile containers with a volume of 5–15 mL. All samples were stored at −70° C.

Both oropharyngeal and fecal samples were collected at two time points: upon admission to the hospital and 7 days later. Some patients also had samples collected 14 days after admission.

#### DNA extraction

The isolation of nucleic acids was conducted using the MagMAXMicrobiome Ultra Nucleic Acid Isolation Kit, with bead tubes and the KingFisherPurification System (Thermo Fisher Scientific, USA), in accordance with the instructions provided by the manufacturer. The DNA was subsequently quantified on Qubit 4 fluorometer by Quant-iT dsDNA BR Assay Kit (Thermo Fisher Scientific, USA).

#### Targeted AR gene panel design

A panel of antibiotic resistance genes potentially found in the human microbiome was constructed using the open GA database MEGARes 2.0.^8^ The panel size was 4,937 regions with a total length of 4,803,602 base pairs. All target sequences were bacterial genes and did not overlap with human genes. The panel of probes for target antibiotics resistance (AR) genes was synthesized by Roche Diagnostics (Madison, USA) based on the target sequences we provided. Panel validation and testing were also conducted by Roche Diagnostics.

#### Targeted AR gene panel sequencing

One hundred nanograms of the extracted DNA was used for library preparation using the KAPA HyperPlus Kit (Roche, Switzerland) and KAPA HyperExplore MAX (Roche, Switzerland) according to the manufacturer’s instructions. The protocol included the following steps: library preparation, hybridization, bead capture, washing, amplification enrichment QC, sequencing, and pre- and post-capture multiplexing. The library underwent a final cleanup using the KAPA HyperPure Beads (Roche, Switzerland) after which the library size distribution and quality were assessed using a high sensitivity DNA chip (Agilent Technologies). Libraries were subsequently quantified by Quant-iT DNA Assay Kit, High Sensitivity (Thermo Fisher Scientific). High-throughput sequencing of the obtained libraries was performed on MGI DNBSEQ-G400 platform (2 × 150 bp paired-end sequencing) using MGIEasy Universal Library Conversion Kit (App-A), High-throughput Sequencing Primer Kit-C (App-C), DNBSEQ-G400RS High-throughput Rapid Sequencing Kit (FCS PE100) and DNBSEQ-G400RS Rapid Sequencing Flow Cell (FCS) according to the manufacturer’s protocol.

The resulting dataset was deposited in NCBI under the BioProject accession number PRJNA1005621.

#### 16S rRNA gene sequencing

Library preparation was performed according to the 16S Metagenomic Sequencing Library Preparation Illumina protocol. Briefly, the extracted DNA was amplified using the 341F and 801R primers, which are complementary to the V3-V4 region of the 16S rRNA gene and contain 5′-Illumina adapter sequences. During the next step, individual amplicons were PCR–indexed and pooled. DNA libraries were sequenced with the MiSeq instrument (Illumina, USA) using the MiSeq reagent kit v3 (Illumina, USA).

#### 16S rRNA amplicon sequencing data

Sequencing reads for 16S rRNA gene amplicons have been deposited in NCBI BioProject under project name PRJNA989180.

### Quantification and statistical analysis

#### Targeted AR gene panel data processing and analysis

The full scheme of AR gene panel bioinformatics data processing and analysis is shown in Figure S1.

The remaining adapters were excised, and quality filtering of the reads was conducted using Trimmomatic (v.0.39–2)^41^ with the following parameters: *SLIDINGWINDOW:10:20, LEADING:20, TRAILING:20, MINLEN:75*.

To eliminate potential contaminant sequences and samples with a low abundance of target genes, the reads were aligned to the human genome assembly GTCh38 (hg38) using bowtie2 (v2.4.2) with the default parameters, i.e., end-to-end alignment, “sensitive” mode, and ambivalent mapping allowed. Reads that mapped to hg38 were excluded from subsequent analysis. In oropharyngeal samples, the proportion of human sequences ranged from 0.8% to 92.4%, with a median value of 14.3%. In fecal samples, the proportion of human sequences ranged from 0% to 11.5%, with a median value of 0.4%. Ten oropharyngeal samples containing more than 70% of human sequences were excluded from subsequent analysis.

The remaining reads that did not map to the human genome were then mapped to the reference database of 4,937 antibiotic resistance (AR) determinants. Ambivalent reads that mapped simultaneously to sequences of different AR gene groups (e.g., beta-lactamases and chloramphenicol acetyltransferases) were removed. Ambivalent reads that simultaneously mapped to different AR genes (e.g., *ermF* and *ermB*) were also excluded to avoid confusion.

Non-ambivalent mappings of sequencing reads to the 4,937 target AR determinant sequences were employed for subsequent analysis.

Upon evaluating AR gene coverage values, one fecal sample was identified as exhibiting inadequate AR gene coverage width, despite satisfactory AR gene read mapping counts. This sample was thus excluded from further analysis.

For each target AR gene sequence, the coverage width was calculated as the proportion of nucleotides covered with at least one read to the total sequence length. Coverage width was set at a 99% threshold. A gene was considered fully covered and present in a given sample if at least one of its sequence variants was covered with reads at 99% of its width. Only fully covered sequences were used in identifying the “core resistome”. Oropharyngeal and fecal samples were analyzed separately.

Genes and sequence variants of genes identified in at least 80% of samples in each biotope were considered to constitute the “core” set of genes. We have also identified “biotope-specific” variants of “core” genes that can be considered “core” in both biotopes. These are defined as specific sequence variants that occur in more than 50% of samples in one biotope and are present in less than 50% of samples in the other. Differences between the frequencies of the core sequence variants of AR genes present in oropharyngeal and fecal samples were assessed using the Chi-square test, and *p*-values were adjusted using the Bonferroni correction.

Diversity scores for each sample were determined by assessing the number of genes that had at least one sequence variant fully covered (≥99 % coverage width) with reads. Diversity scores were then compared between the different datasets using the Mann-Whitney U test.

For each of the 646 genes presented as 4,937 sequence variants in the reference database, coverage depth was calculated using modified reads per kilobase per million mapped reads (RPKM) and transcripts per million mapped reads (TPM) metrics. RPKM values were calculated according to the following formula:

(Equation 1) $$\text{RPK}M_{\text{gene}}=\frac{\text{reads}\;\text{mapped}\;\text{to}\;\text{all}\;\text{sequence}\;\text{variants}}{\text{total}\;\text{reads}\;\text{in}\;\text{sample}\cdot \text{median}\;\text{sequence}\;\text{variant}\;\text{lengh}\;\text{of}\;a\;\text{gene}}$$

In this calculation, the total number of reads in a sample is counted in millions, while the median sequence variant length is counted in kilobases.

Similarly, TPM values were calculated as follows:

(Equation 2) $$\text{TP}M_{\text{gene}}=10^{6}\cdot \frac{\frac{\text{reads}\;\text{mapped}\;\text{to}\;\text{all}\;\text{sequence}\;\text{variants}}{\text{median}\;\text{sequence}\;\text{variant}\;\text{length}\;\text{of}\;a\;\text{gene}}}{\sum_{\frac{\text{reads}\;\text{mapped}\;\text{to}\;\text{all}\;\text{sequence}\;\text{variants}}{\text{median}\;\text{sequence}\;\text{variant}\;\text{length}\;\text{of}\;a\;\text{gene}}}}$$

These metrics are similar to those used in RNA-seq data processing, with the exception that high variability of target genes was considered and the sum of reads mapped to each sequence variant of a target gene was divided by the median length of sequence variants reported for each target gene.

In the subsequent analysis, RPKM values were predominantly employed, with the exception of the analysis of gene abundance changes over time, where both RPKM and TPM values were used.

To evaluate the dissimilarity of fecal and oropharyngeal resistomes, we calculated Bray-Curtis distances based on RPKM values obtained for each gene and each sample. Principal coordinates analysis (PCoA) was conducted based on Bray-Curtis dissimilarities using the capscale method from the vegan R package (version 2.6–4).

Additionally, Bray-Curtis distances were calculated for each biotope within the patient cohort for whom samples were collected at both time points. For each sample, the “closest” sample with the minimal Bray-Curtis distance was assigned. The proportion of samples within each biotope where the sample with the minimal Bray-Curtis distance was the paired sample (i.e., a sample taken from the same patient and the same biotope at a different time point) was then calculated.

A Dirichlet-multinomial mixture model was employed to perform clustering based on RPKM values of all samples. Clustering was performed with the DirichletMultinomial R package (version 1.40.0).

To identify potential resistotypes, we have performed DMM clustering within each biotope. Model fit was evaluated using Laplace, BIC and AIC metrics. The optimal number of clusters was determined based on the number of Dirichlet components corresponding to lower values of all the three metrics.

The AR gene compositions of the samples belonging to different resistotypes were compared using the DESeq2 package in R based on the mapped read counts for each of the genes. The sums of reads that mapped to all sequence variants of each gene were utilized. Genes that accounted for more than 30% of samples with nonzero values were included in the analyses. The results were filtered by *p*-value, with only significant entries with an adjusted *p*-value ⟨< 0.05 being used for further analysis.

To detect differences in AR gene abundance between different time points, we used samples that were collected at two time points: at the time of the admission, and one week later. The Wilcoxon matched-pairs signed-rank test was performed using RPKM and TPM values. To compensate for multiple comparisons, the Bonferroni correction was employed. A gene was deemed to be significantly overrepresented at either time point if it was identified using both RPKM and TPM metrics with adjusted *p*-values ⟨< 0.05.

Within each biotope and resistotype, Spearman’s rank correlation coefficients were calculated for each pair of genes based on their RPKM values. Correlation coefficients were only calculated for those genes that had non-zero RPKM values in more than 50% of the samples. P-values were adjusted using the Bonferroni correction. Genes were considered co-abundant, if their correlation coefficient was greater than 0.9 and adjusted *p*-values were less than 0.05. Adjacency matrices of co-abundant genes were then calculated.

#### 16s rRNA gene data processing and analysis

The full scheme of the bioinformatics processing and analysis of the 16S rRNA gene amplicon sequencing data is shown in Figure S2.

The remaining adapters were removed using Trimmomatic v0.36,^41^ and quality filtering of the reads was performed using the filterAndTrim function from the DADA2 package.^42^

Denoising, merging and chimera removal were carried out with the DADA2 v1.24.0 software with the following parameters: *learnErrors: nbases=1e+09, randomize=TRUE, MAX_CONSIST=2, dada: pool = TRUE, mergePairs: minOverlap=18, removeBimeraDenovo: allowOneOff=FALSE, method=*“*consensus”*.

Taxonomic annotation was carried out against the SILVA v138 reference database.^43^

Potential contaminant sequences were removed with the “frequency” method using the package decontam^44^ version 1.10.0. Statistical analysis of the decontaminated 16S rRNA gene data was performed in R using vegan and phyloseq^45^ packages.

To identify potential enterotypes, DMM clustering was performed as previously described (see "targeted AR gene panel data processing and analysis" subsection) using genus-level abundance data.

The bacterial composition of the samples belonging to the different enterotypes was compared at the genus, species and amplicon sequence variant (ASV) levels using the DESeq2 package in R.^46^ Only those taxa that accounted for more than 30% of the samples with non-zero values were included in the subsequent analyses. The results were filtered by *p*-value in such a way that only those entries with an adjusted *p*-value ⟨< 0.05 were used in further analyses.

Bacterial diversity was estimated at the species level using the Chao1 index, a non-parametric measure that estimates total species richness by accounting for unobserved species based on rare species data.^47^ The Chao1 index was calculated using the vegan package. Additionally, the number of the observed genera was estimated as an additional diversity metric. Diversity values were compared between different sets of samples using the Mann-Whitney U test and the Kruskal-Wallis rank-sum test.

To assess sample dissimilarity, Bray-Curtis distances were calculated for samples within each biotope using the vegan package in R.

#### Comparing resistome and taxonomy data

We used taxonomy data obtained via 16S rRNA amplicon sequencing at the species and genus levels and have calculated Spearman’s rank correlation coefficients of each pair of a taxon and an AR gene. Only genes and taxa with a minimum of 50% non-zero values across samples were included in the analysis. The *p*-values obtained were subjected to a Bonferroni correction. Only correlations with a coefficient of 0.5 or greater or less than −0.5 were subjected to analysis.

We have also calculated Spearman’s rank correlation coefficients for the number of AR genes and AR gene sequence variants identified in each sample and Bray-Curtis distances between samples taken from the same patients at different time points based on genus abundance data. Additionally, the Mann-Whitney U test was employed to ascertain whether there was a statistically significant difference in the number of AR genes at the initial time point between patients who exhibited a change in enterotypes during the first week of therapy and those who did not.

The bacterial composition of the samples belonging to different resistotypes was compared using the DESeq2 package in R. Only taxa that accounted for more than 30% of the samples with nonzero values were included in the analyses. To account for multiple comparisons, the Benjamini and Hochberg correction method was employed. Only significant entries with adjusted *p*-values ⟨< 0.05 were used in the analysis.

## Supplemental information

Document S1. Figures S1–S14, Tables S2–S7, S10–S12, S15, and S16

Table S1. Oropharyngeal and fecal samples collected from patients in different time points, related to Table 2

Table S8. Minimal Bray-Curtis distances for each of the paired fecal and oropharyngeal samples, based on AR genes RPKM counts, related to Figure 4

Table S9. Fecal resistotypes and enterotypes assigned to each patient in different time points with additional metadata and antibiotics intake in the hospital, related to Figures 10, 18, and 22

Table S13. Pairs of co-abundant oropharyngeal AR genes identified as a result of Spearman correlation tests based on RPKM values, related to Figure 14

Table S14. Minimal Bray-Curtis distances for each of the paired fecal and oropharyngeal samples, based on genera counts obtained with 16s rRNA gene amplicon sequencing, related to Figure 16
