# Supplementary material for: Oropharyngeal resistome remains stable during COVID-19 therapy, while fecal resistome shifts toward a less diverse resistotype
Source: iScience. 2024 Nov 5;27(12):111319. doi: 10.1016/j.isci.2024.111319 (PMC11617248; doi:10.1016/j.isci.2024.111319)
Supplement: Document S1. Figures S1–S14, Tables S2–S7, S10–S12, S15, and S16 [file mmc1.pdf]

iScience, Volume *27*

Supplemental information

Oropharyngeal resistome remains stable during COVID-19 therapy, while fecal resistome shifts toward a less diverse resistotype

Elizaveta V. Starikova, Yulia S. Galeeva, Dmitry E. Fedorov, Elena V. Korneenko, Anna S. Speranskaya, Oksana V. Selezneva, Polina Y. Zoruk, Ksenia M. Klimina, Vladimir A. Veselovsky, Maxim D. Morozov, Daria I. Boldyreva, Evgenii I. Olekhnovich, Alexander I. Manolov, Alexander V. Pavlenko, Ivan E. Kozlov, Oleg O. Yanushevich, Natella I. Krikheli, Oleg V. Levchenko, Dmitry N. Andreev, Filipp S. Sokolov, Aleksey K. Fomenko, Mikhail K. Devkota, Nikolai G. Andreev, Andrey V. Zaborovsky, Sergei V. Tsaregorodtsev, Vladimir V. Evdokimov, Petr A. Bely, Igor V. Maev, Vadim M. Govorun, and Elena N. Ilina

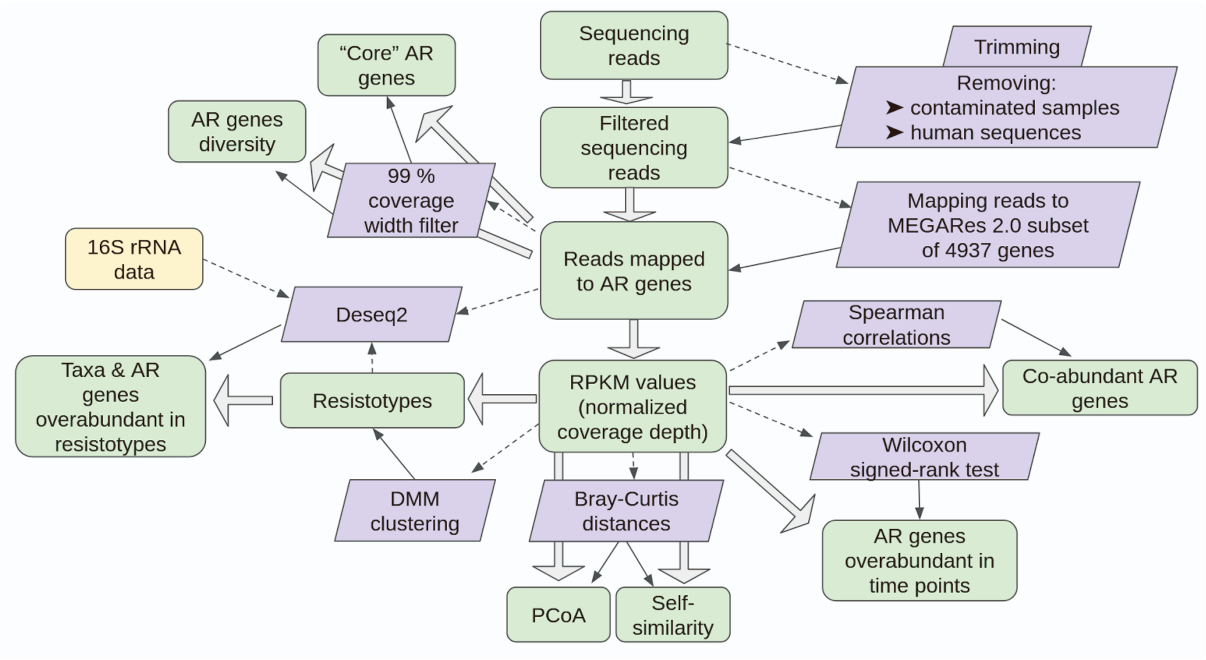

Figure S1. AR gene sequencing panel bioinformatics analysis scheme. **Related to STAR methods section**

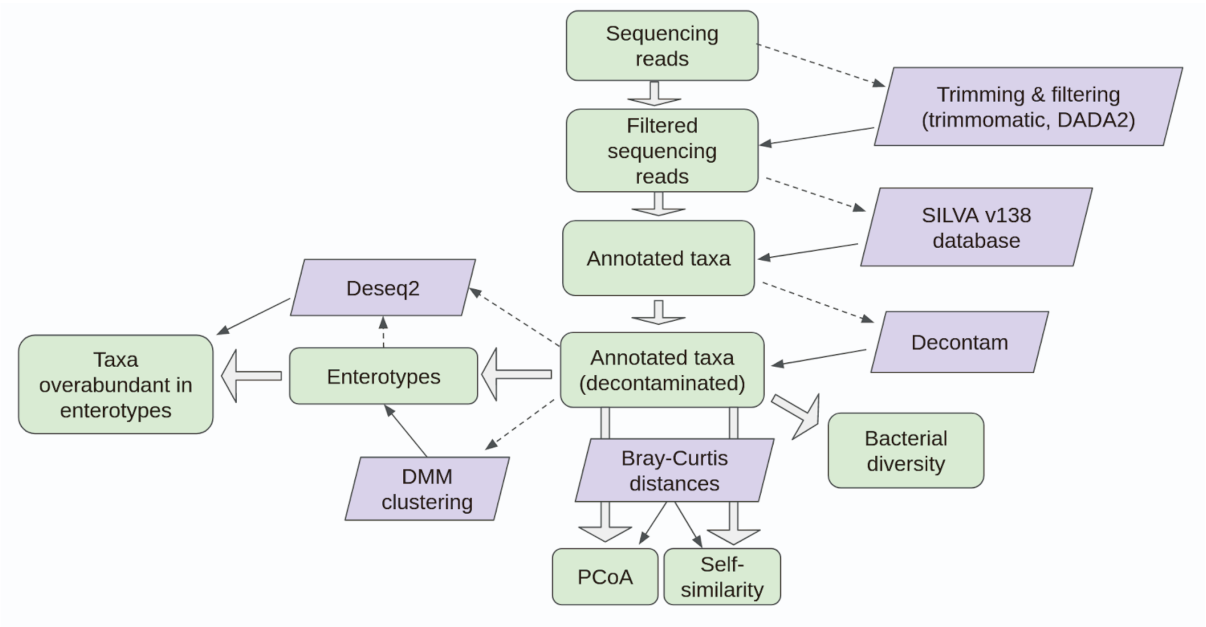

Figure S2. 16S rRNA gene amplicon sequencing data bioinformatics analysis scheme. **Related to STAR methods section**

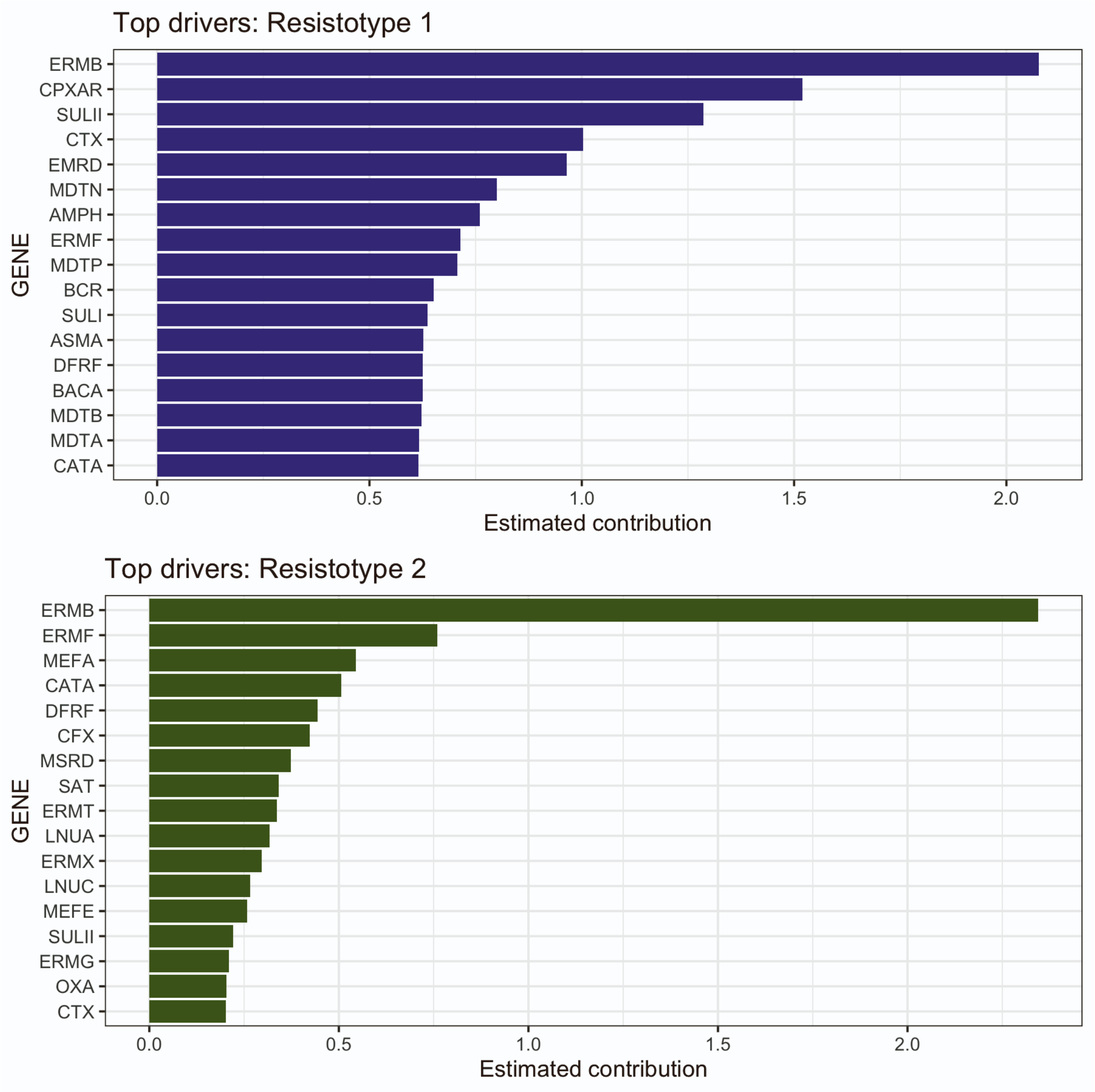

Figure S3. Top AR gene drivers contributing to resistotypes 1 and 2. **Related to Figure 6**

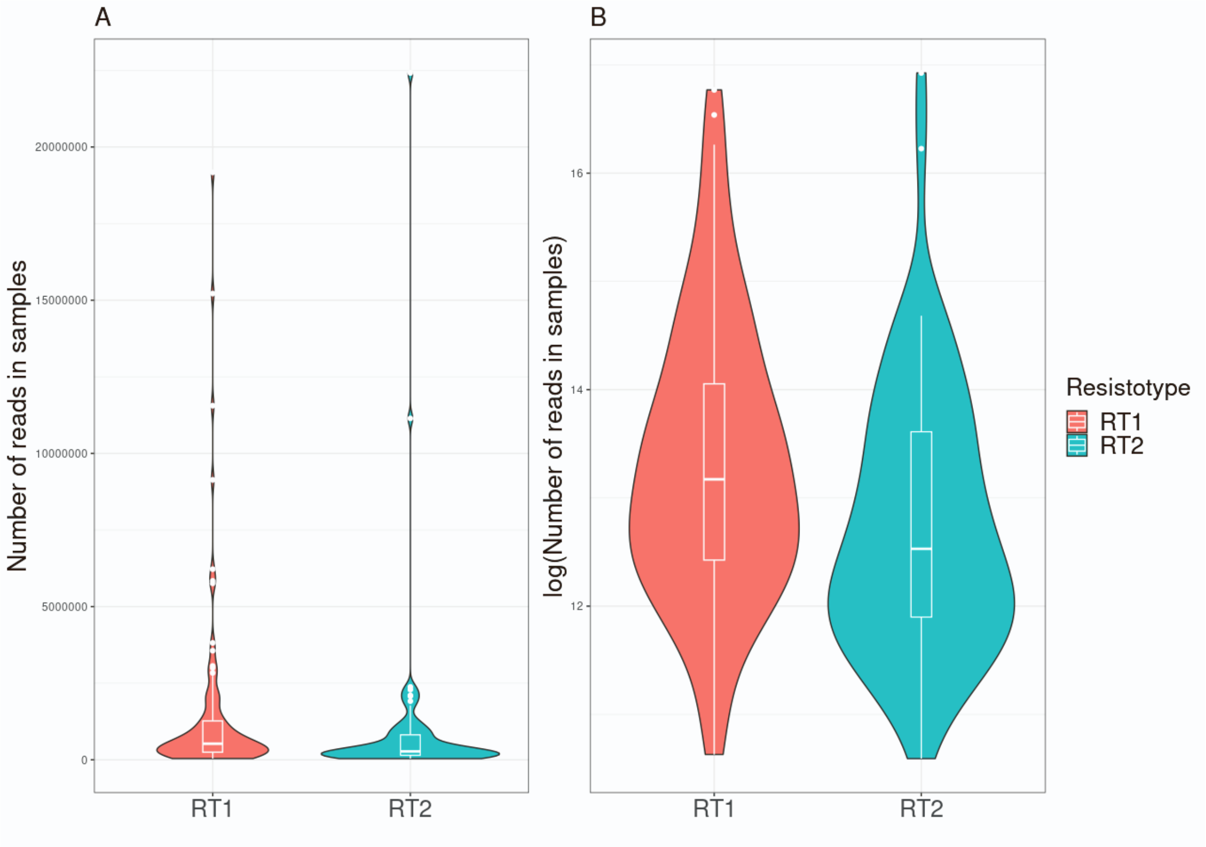

Figure S4. Boxplots/violin plots illustrating distribution of numbers of sequencing reads in the samples (grouped by assigned resistotypes). (A) Raw counts of sequencing reads (B) Log-transformed counts of sequencing reads. **Related to Figure 9**

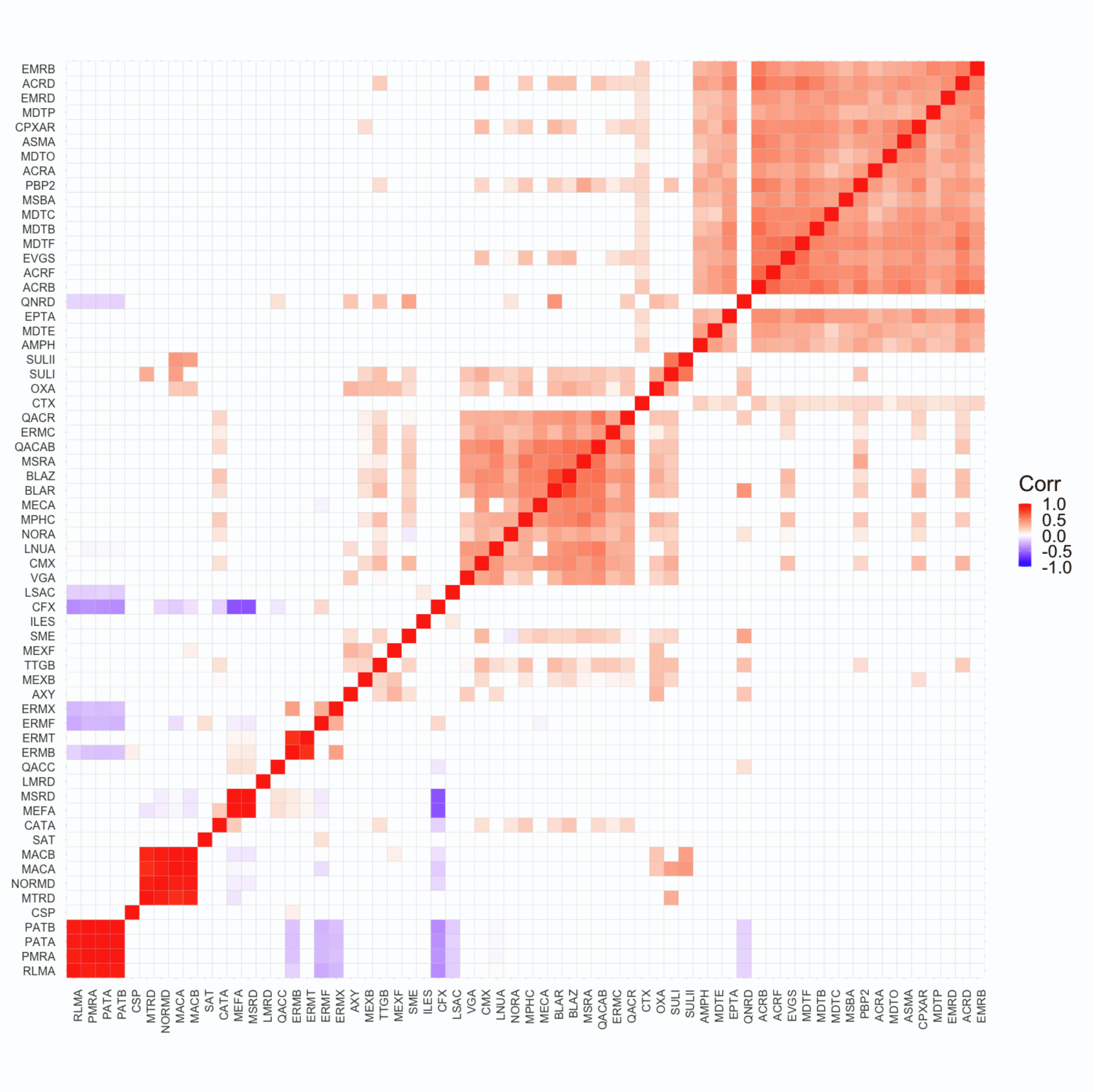

Figure S5. Heatmap representing correlation matrix of AR genes identified in oropharyngeal samples. Insignificant correlations (p < 0.05) are blanked out. Red and blue colors indicate positive and negative Spearman correlations, correspondingly. **Related to Figure 13**

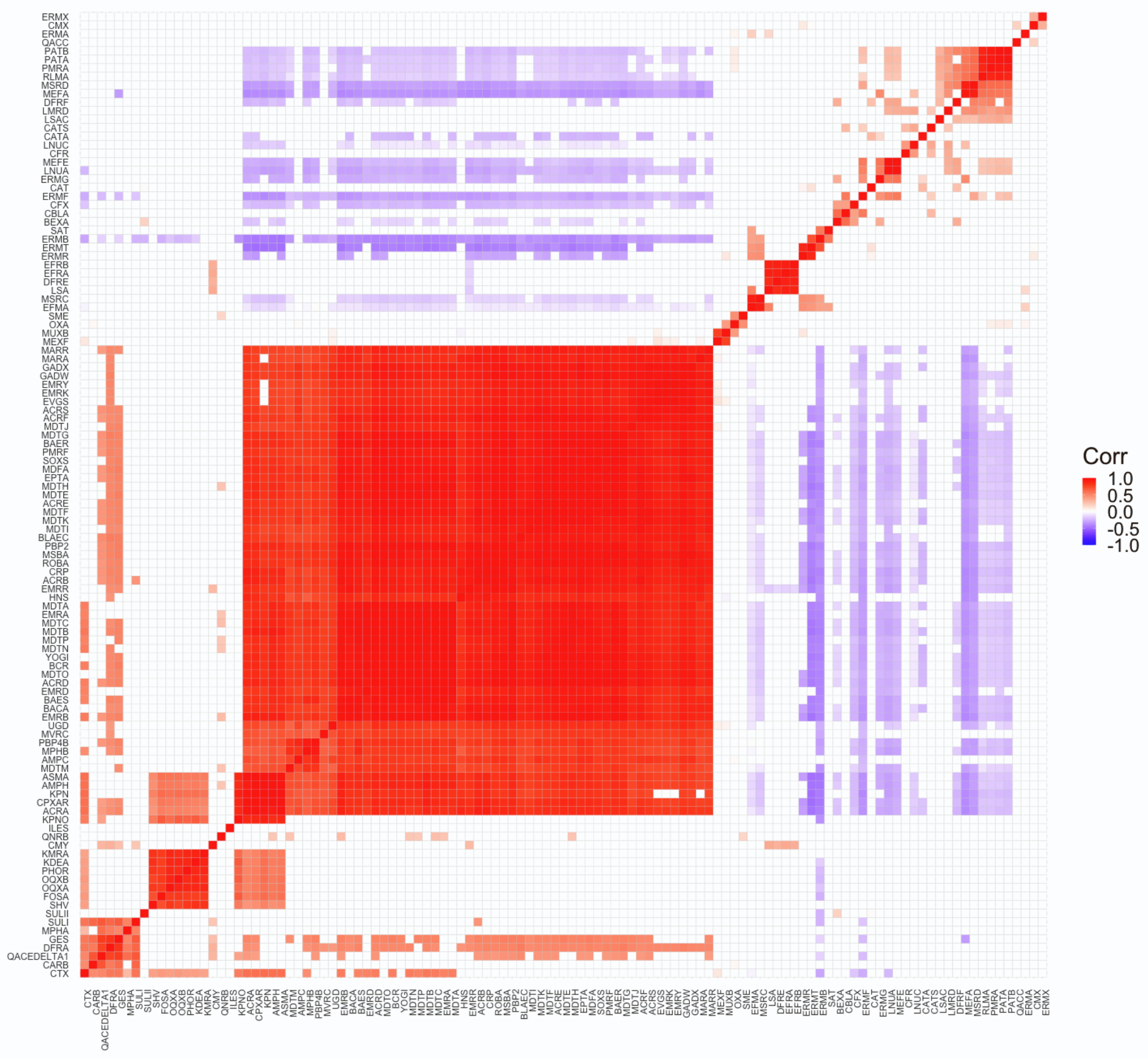

Figure S6. Heatmap representing correlation matrix of AR genes identified in fecal samples. Insignificant correlations ($p < 0.05$) are blanked out. Red and blue colors indicate positive and negative Spearman correlations, correspondingly. **Related to Figure 14**

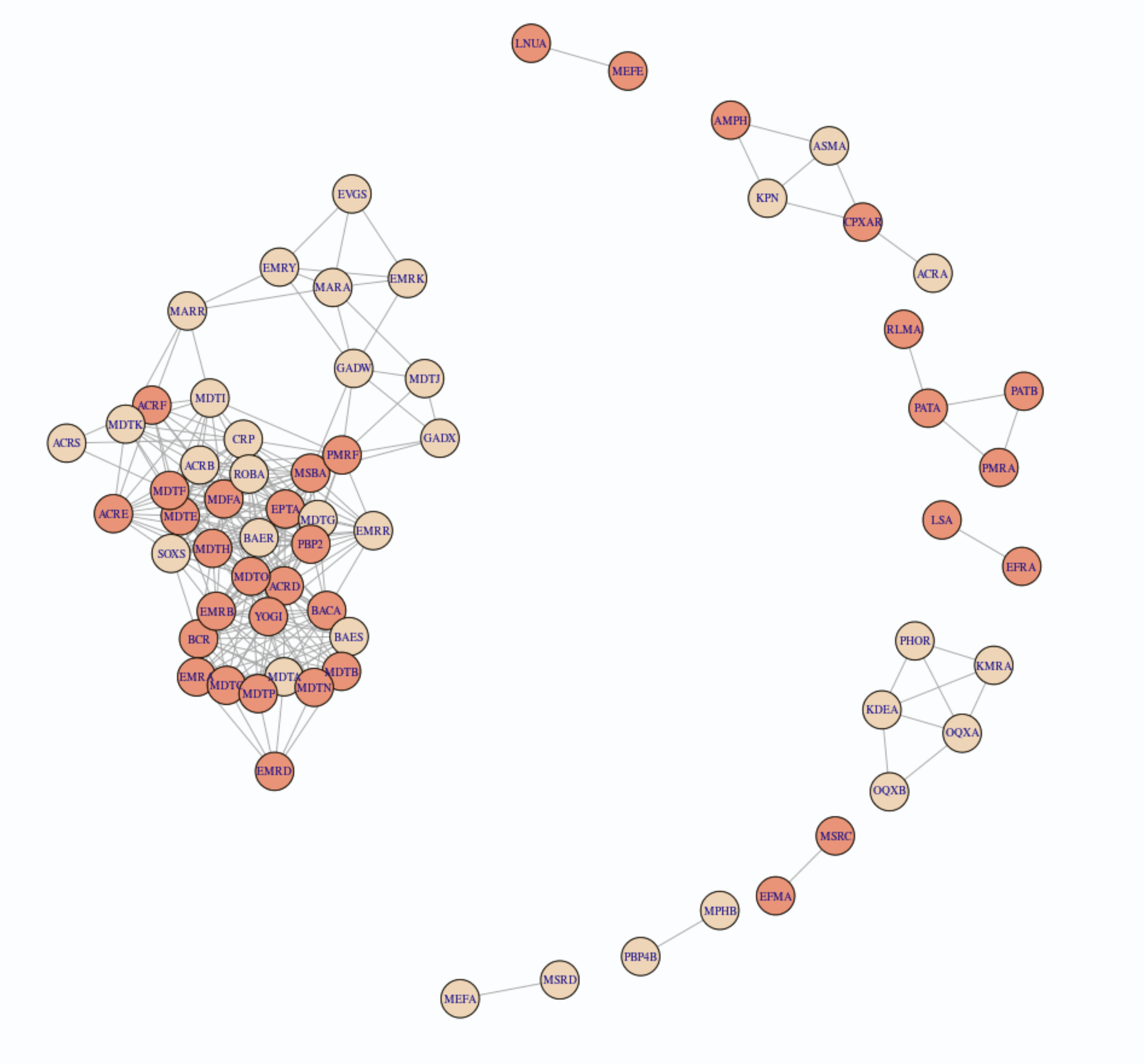

Figure S7. Gene clusters showing high (>0.9) correlation coefficients based of RPKM values in fecal samples of Resistotype 1 (RT1). Pink color represents vertices that are also found in co-abundant gene clusters of fecal resistotype 2 (RT2). **Related to Figure 14**

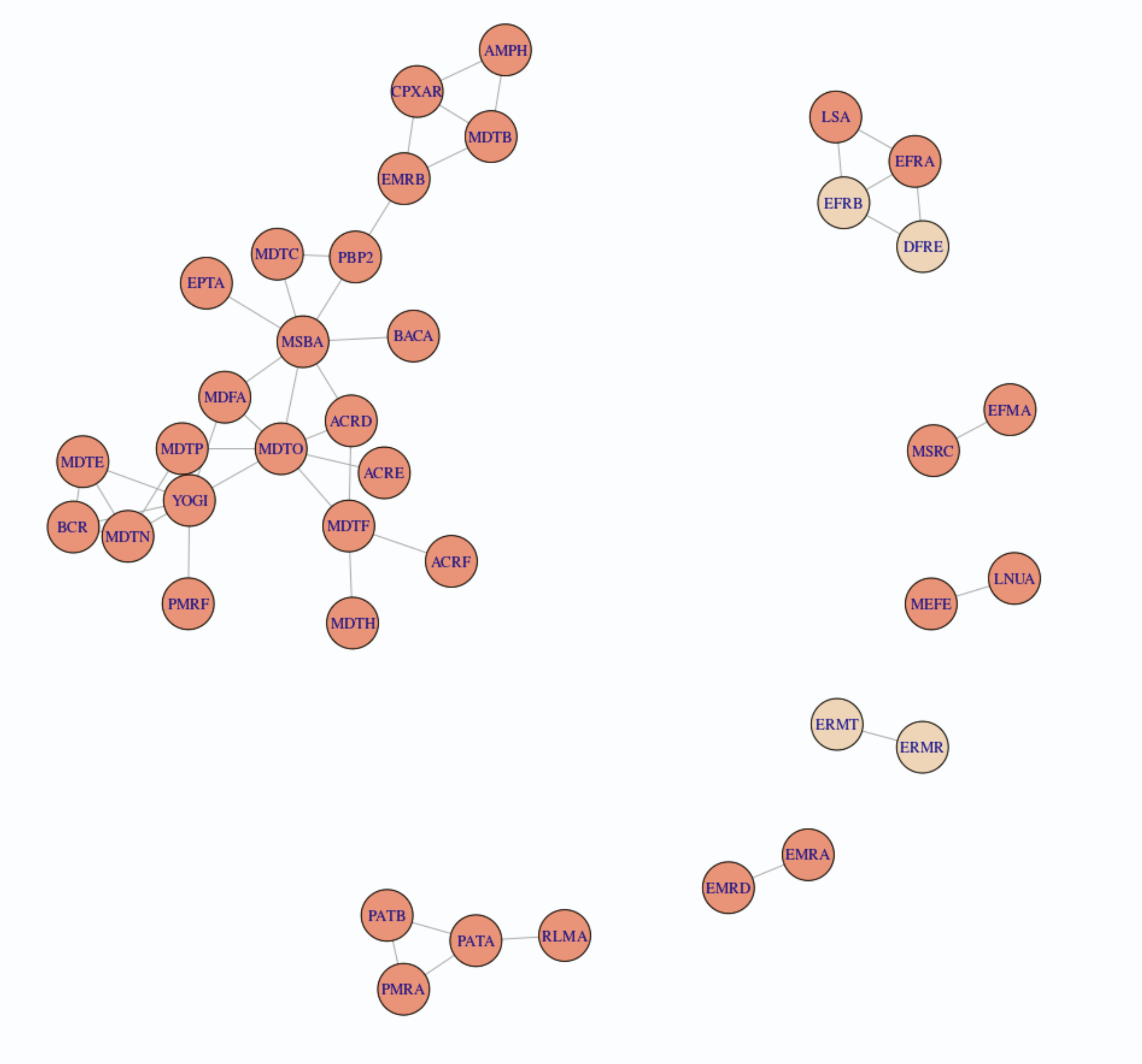

Figure S8. Gene clusters showing high (>0.9) correlation coefficients based of RPKM values in fecal samples of Resistotype 2 (RT2). Correlation was estimated as Spearman's rank correlation coefficient. Pink color represents vertices that are also found in co-abundant gene clusters of fecal resistotype 1 (RT1). **Related to Figure 14**

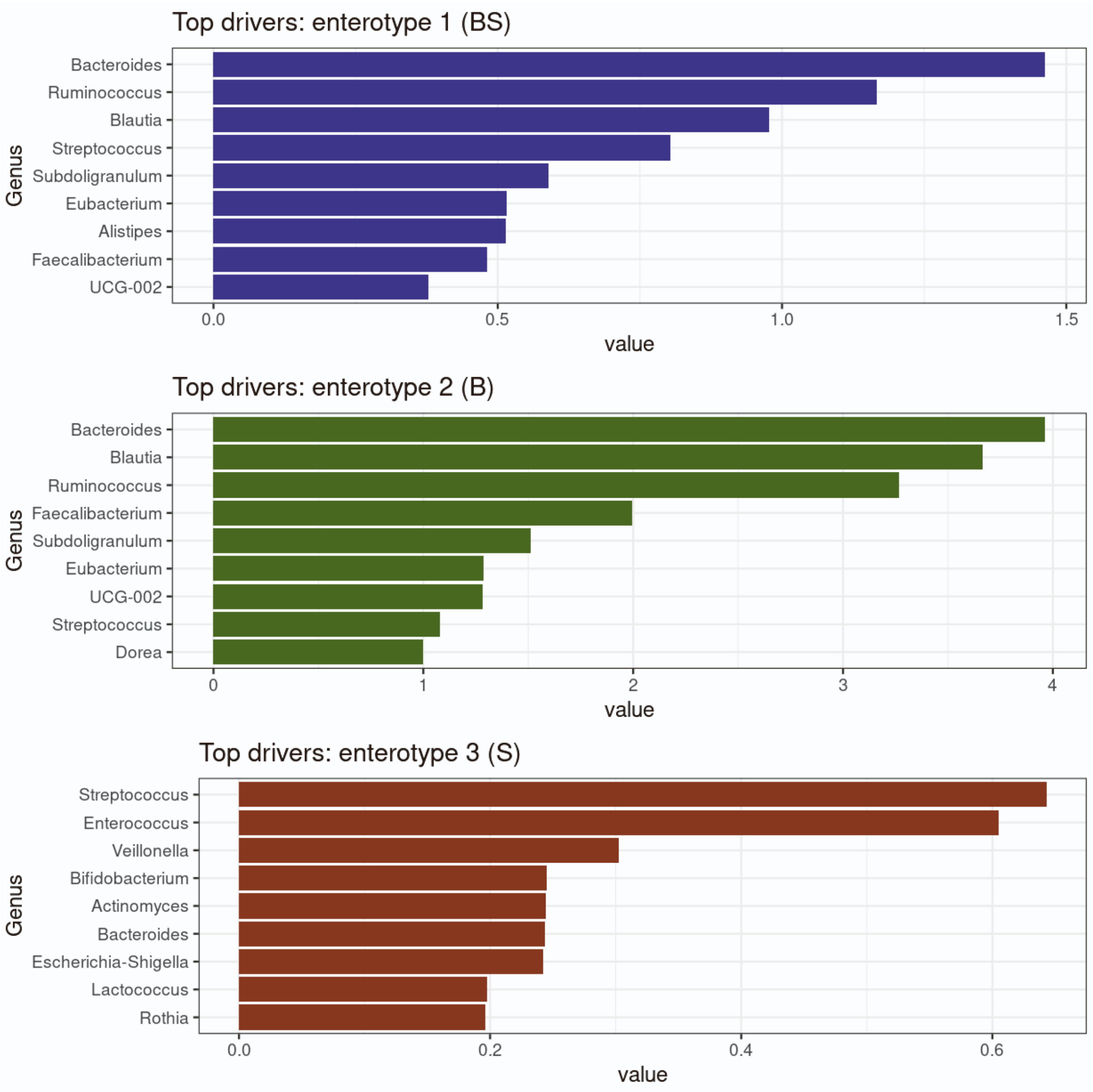

Figure S9. Barplots illustrating top drivers contributing to the three identified enterotypes. **Related to Figure 17**

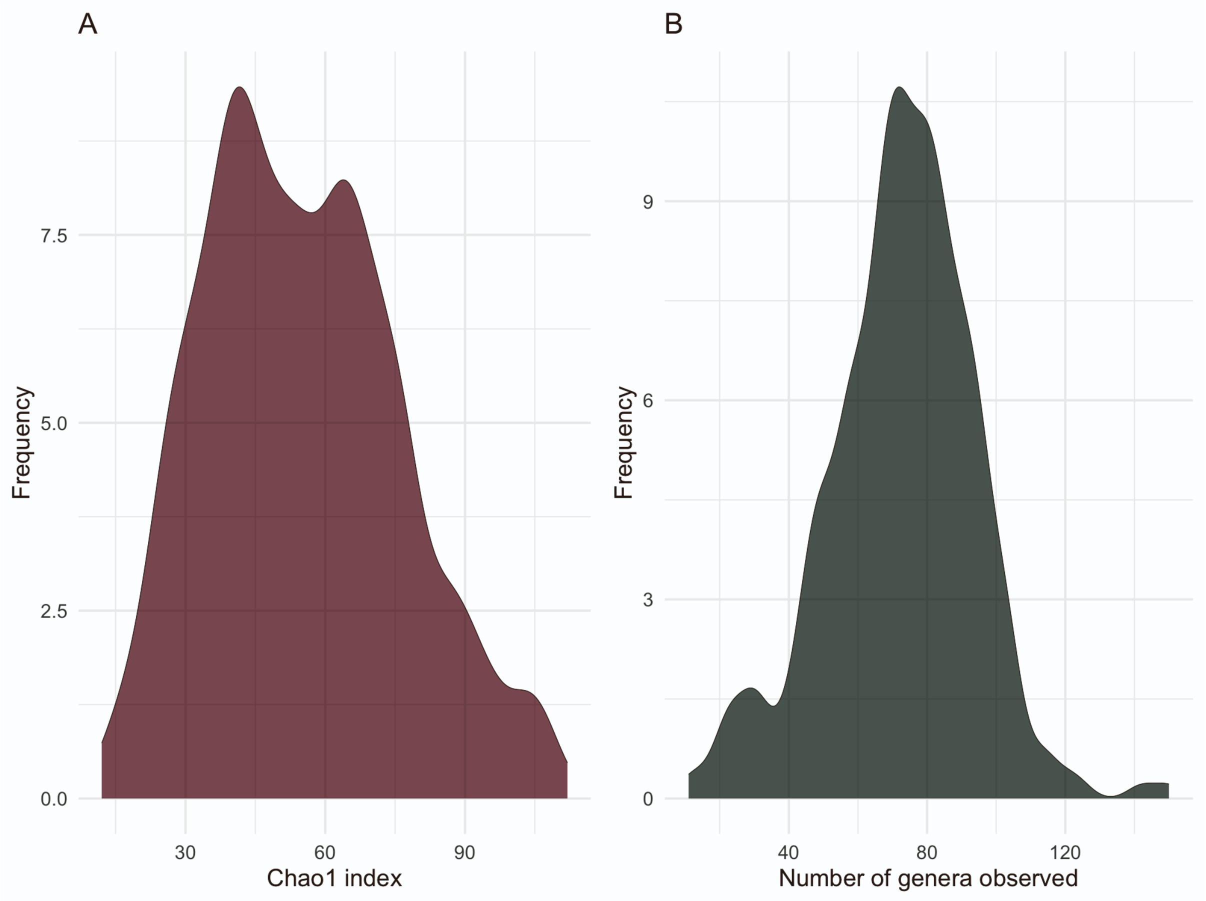

Figure S10. Bacterial diversity indices calculated for the oropharyngeal samples. (A) Species level, Chao1 index. Median 54, sd 20.99. (B) Genus level, the number of the observed genera. Median 73, sd 21.3. **Related to Figure 19**

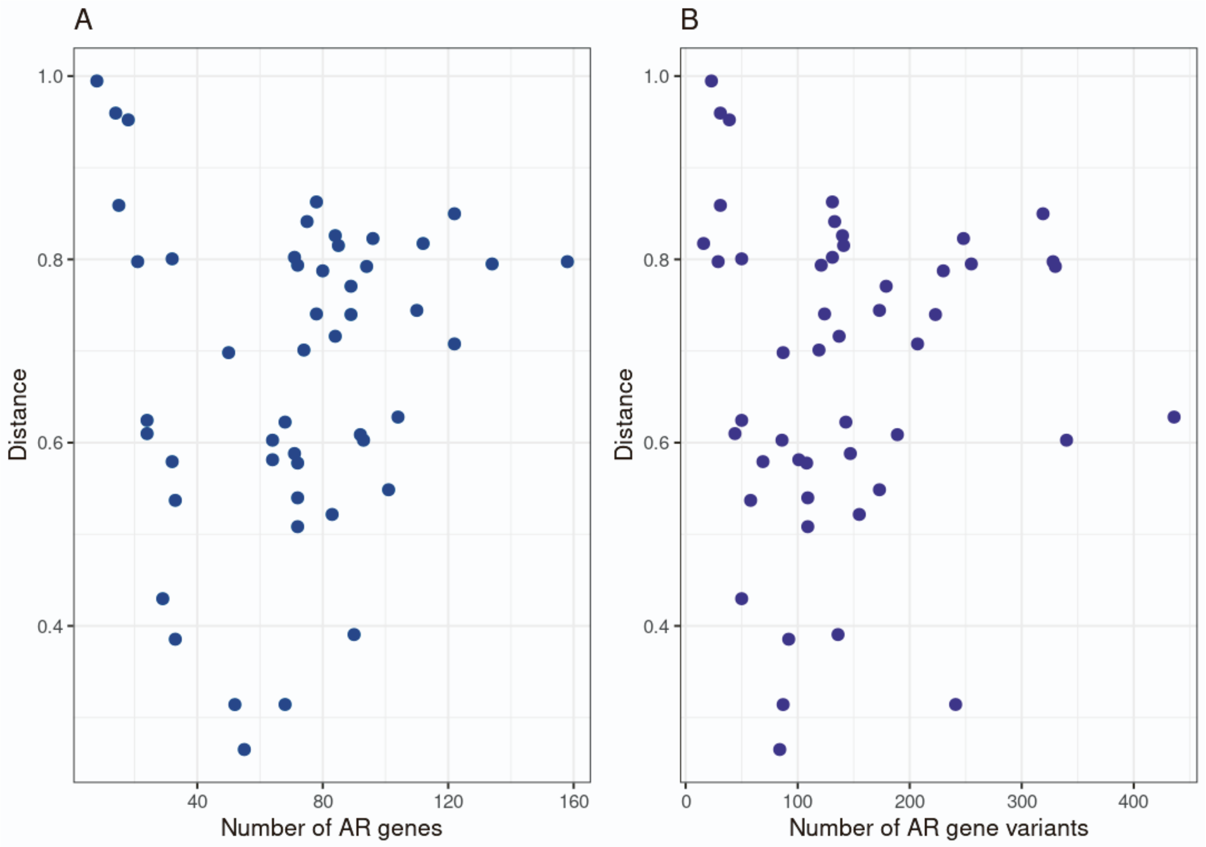

Figure S11. Scatter plots representing Bray-Curtis distances of the fecal samples taken in different time points for the same patient as a function of (A) the initial number of AR genes in the sample (B) the initial number of AR gene variants in the sample. **Related to Figure 3**

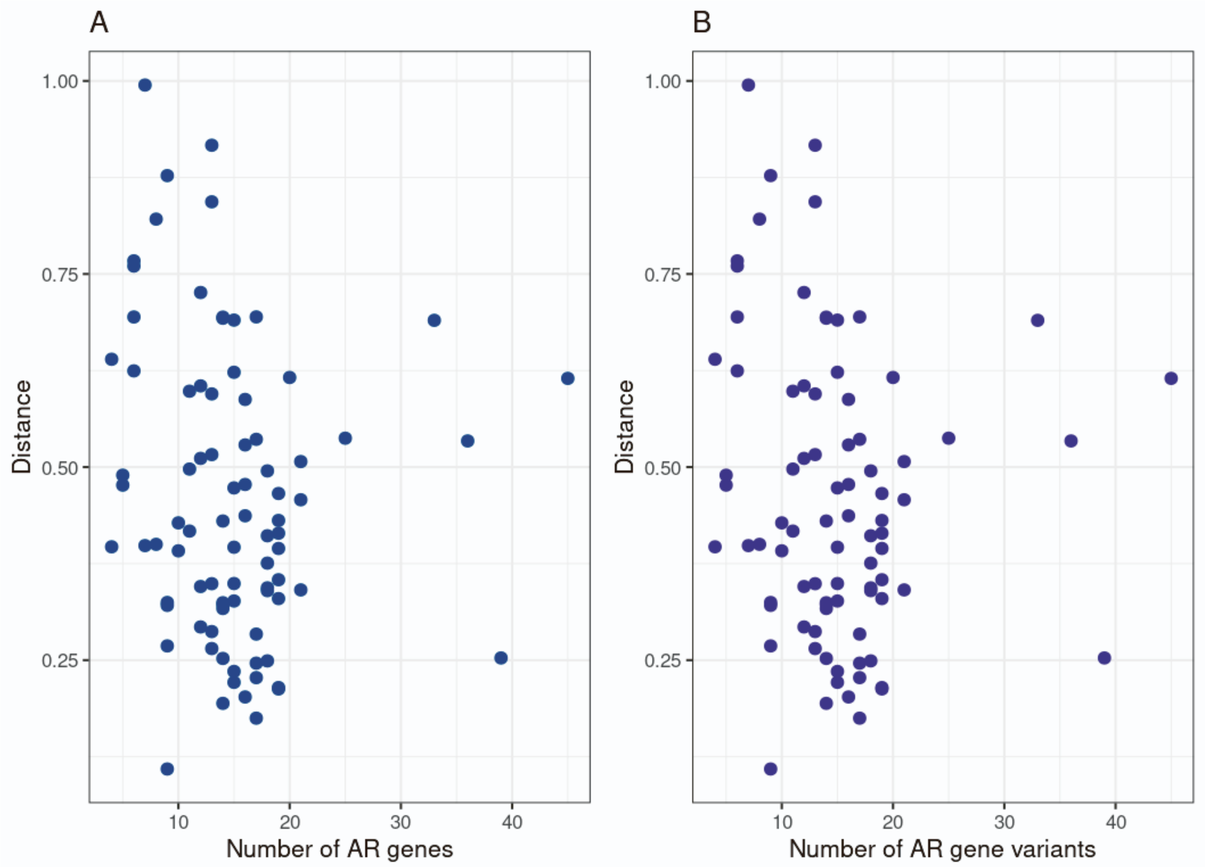

Figure S12. Scatter plots representing Bray-Curtis distances of the oropharyngeal samples taken in different time points for the same patient as a function of (A) the initial number of AR genes in the sample (B) the initial number of AR gene variants in the sample. **Related to Figure 3**

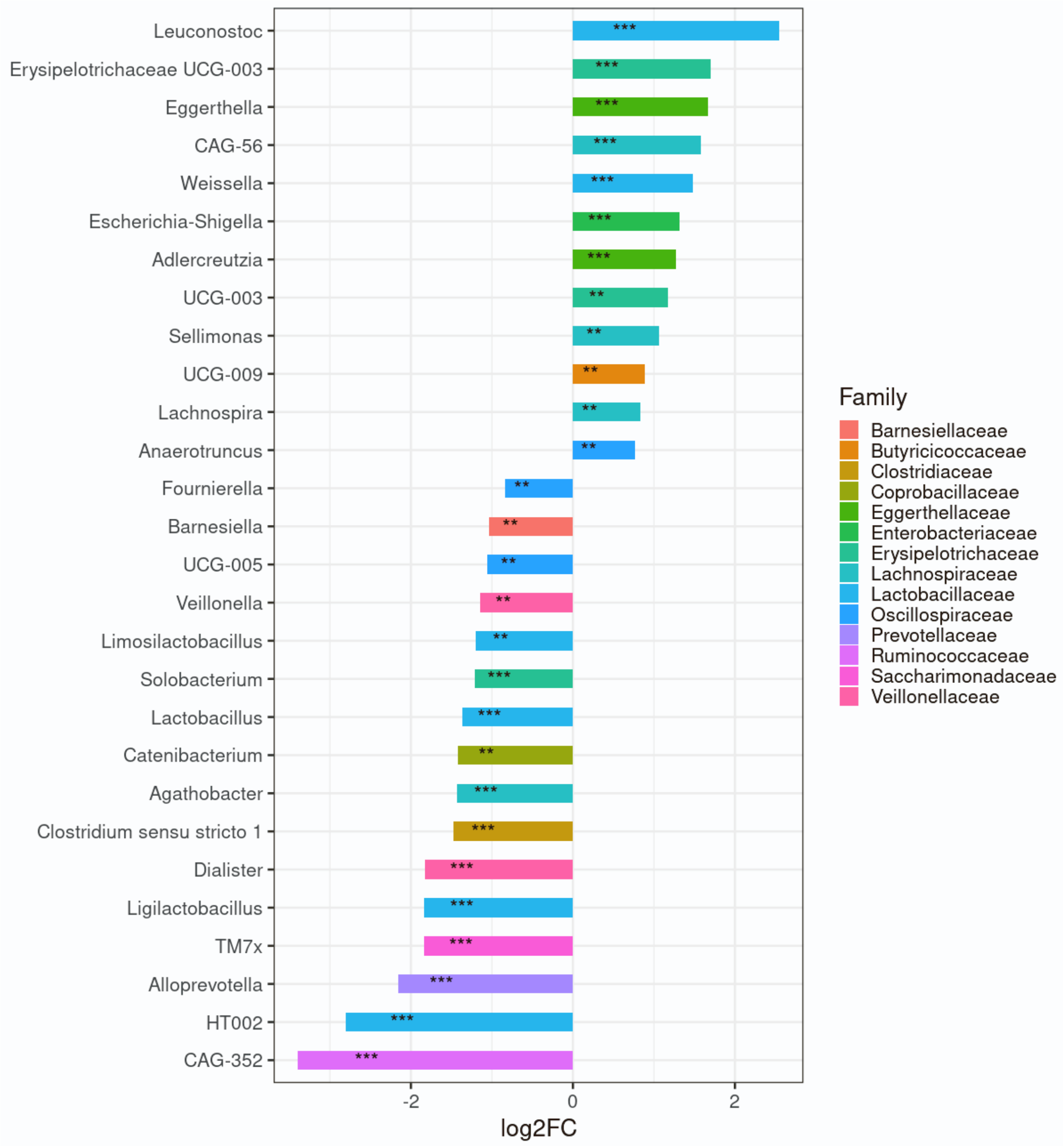

Figure S13. Barplots illustrating log2 fold change of genus counts between resistotypes 1 and 2 calculated during DESeq2 analysis. Bars oriented right (positive log2FC) correspond to genera overrepresented in RT2, bars oriented left (negative log2FC) correspond to genera overrepresented in RT1. Genera with p-values < 0.05 are marked with “**”, and genera with p-values < 0.01 are marked with “***”. For details, see Supplementary Table S18. **Related to Figure 20**

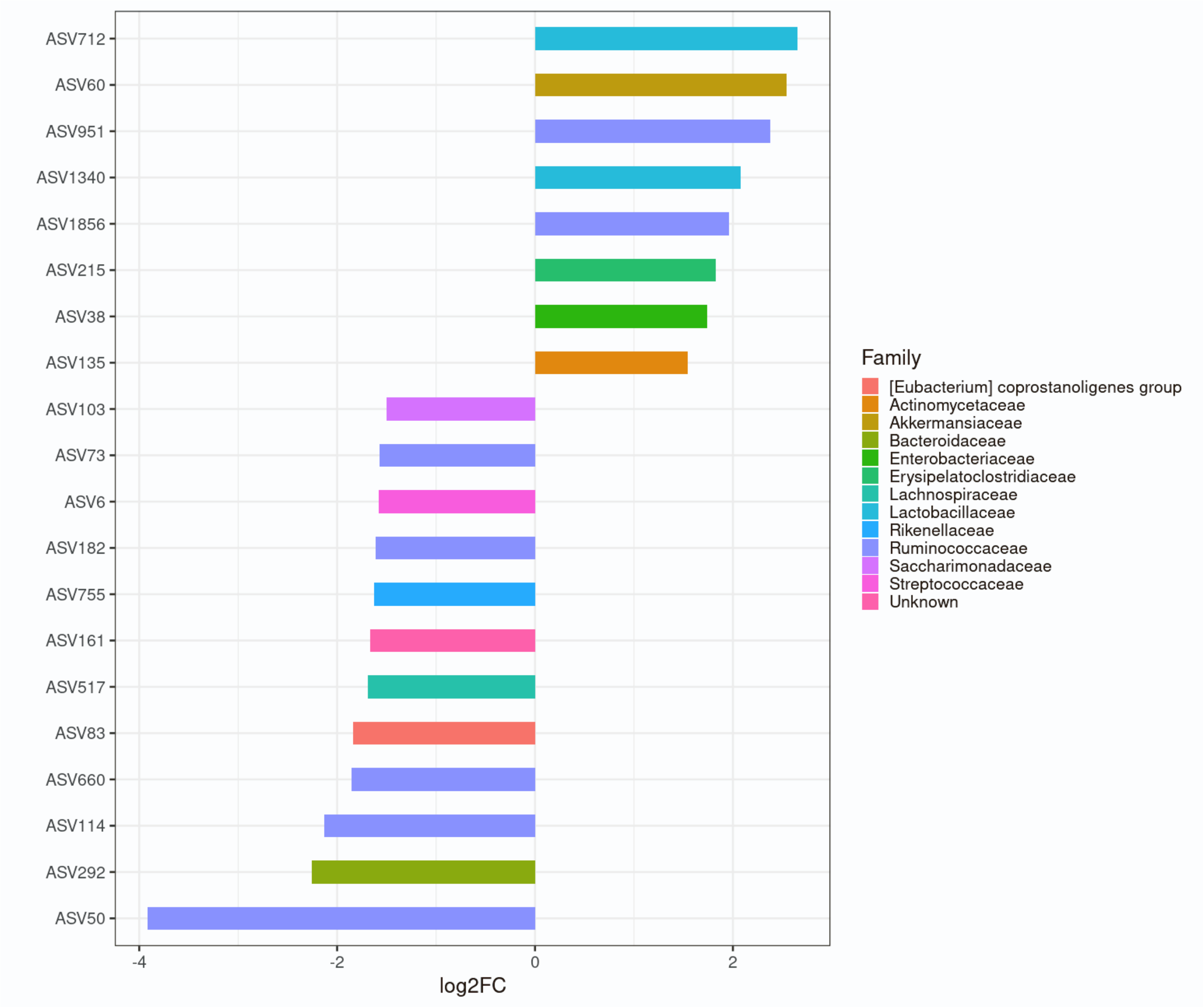

Figure S14. Barplots illustrating log2 fold change of ASV counts between resistotypes 1 and 2 calculated during DESeq2 analysis. Bars oriented right (positive log2FC) correspond to ASVs overrepresented in RT2, bars oriented left (negative log2FC) correspond to ASVs overrepresented in RT1. On this figure, only ASVs with log2 fold change values of more than 1.5 or less than -1.5 are displayed. For the full set of ASVs see Supplementary Table S20. **Related to Figure 20**

Table S2. The numbers of nucleotide sequences in targeted AMR gene sequencing panel presented by antibiotic group. **Related to STAR Methods**

Antibiotic group	Number of nucleotide sequences
Beta-lactams	3603
MLS (macrolides, lincosamides, and streptogramines)	250
Quinolone	186
Phenicol	105
Trimethoprim	100
Sulfonamides	64
Phosphomycin	52
Metronidazole	17
Rifampin	17
Nucleosides	15
Antimicrobial peptides	11
Fusidic acid	8
Bacitracin	6
Mupirocin	4
Aminocoumarins	2
Thiopeptides	2
Elfamycin	1
Lipopeptides	1
Tetracenomycin	1
Tetracycline	1
Multiple (multi-drug resistance)	491

Table S3. The numbers of nucleotide sequences in targeted AMR gene sequencing panel presented by antibiotic group and resistance mechanism. **Related to STAR Methods**

**AR determinant classification (resistance mechanism), according to MEGARes 2.0 database**	**Number of sequences**	**AB type**
Aminocoumarin-resistant DNA topoisomerases	1	Aminocoumarins
Aminocoumarin efflux pump	1	Aminocoumarins
Bacitracin ABC transporter	3	Bacitracin
Chloramphenicol acetyltransferase	47	Phenicol
Chloramphenicol hydrolase	2	Phenicol
Chloramphenicol phosphotransferase	4	Phenicol
Class A beta-lactamases	1284	Beta-lactams
Class B beta-lactamases	410	Beta-lactams
Class C beta-lactamases	950	Beta-lactams
Class D beta-lactamases	900	Beta-lactams
Defensin-resistant *mprF*	4	Cationic antimicrobial peptides
Dihydrofolate reductase	100	Trimethoprim
Drug and biocide ABC efflux pumps	3	*Non-specific resistance*
Drug and biocide ABC efflux regulator	1	*Non-specific resistance*
Drug and biocide MATE efflux pumps	17	*Non-specific resistance*
Drug and biocide MATE efflux regulator	1	*Non-specific resistance*
Drug and biocide MFS efflux pumps	59	*Non-specific resistance*
Drug and biocide MFS efflux regulator	10	*Non-specific resistance*
Drug and biocide RND efflux pumps	169	*Non-specific resistance*
Drug and biocide RND efflux regulator	36	*Non-specific resistance*
Drug and biocide SMR efflux pumps	52	*Non-specific resistance*
Drug and biocide SMR efflux regulator	1	*Non-specific resistance*
Drug and biocide and metal MFS efflux pumps	3	*Non-specific resistance*
Drug and biocide and metal RND efflux pumps	25	*Non-specific resistance*
Drug and biocide and metal RND efflux regulator	7	*Non-specific resistance*
Drug and biocide and metal resistance regulator	2	*Non-specific resistance*
Drug and biocide and metal resistance regulator	1	*Non-specific resistance*
Drug and metal MFS efflux pumps	4	*Non-specific resistance*
Edeine acetyltransferase	1	Cationic antimicrobial peptides
Elfamycin efflux pumps	1	Elfamycins
Flavin reductase	1	Sulfonamides
Fluoroquinolone ABC efflux pump	2	Fluoroquinolones
Fluoroquinolone resistance phosphotransferase	1	Fluoroquinolones

Fosfomycin MFS efflux pump	2	Fosfomycin
Fosfomycin phosphorylation	3	Fosfomycin
Fosfomycin thiol transferases	47	Fosfomycin
Fusidic acid esterases	3	Fusidic acid
Lincosamide nucleotidyltransferases	23	MLS
Lipid A modification	4	Cationic antimicrobial peptides
Lipid A phosphatase	1	Lipopeptides
MDR 23s ribosomal RNA methyltransferase	10	*Non-specific resistance*
MDR acetyltransferase	1	*Non-specific resistance*
MDR regulator	7	*Non-specific resistance*
MLS resistance ABC efflux pumps	19	MLS
MLS resistance MFS efflux pumps	26	MLS
Macrolide esterases	15	MLS
Macrolide glycosyltransferases	5	MLS
Macrolide phosphotransferases	27	MLS
Monooxygenase	1	Rifampin
Multi-drug ABC efflux pumps	54	*Non-specific resistance*
Multi-drug MATE efflux pumps	2	*Non-specific resistance*
Multi-drug MFS efflux pumps	8	*Non-specific resistance*
Multi-drug RND efflux pumps	13	*Non-specific resistance*
Multi-drug RND efflux regulator	4	*Non-specific resistance*
Multi-drug SMR efflux regulator	1	*Non-specific resistance*
Mupirocin-resistant isoleucyl-tRNA synthetase	4	Mupirocin
Mutant porin proteins	4	Beta-lactams
Nim nitroimidazole reductase	17	Metronidazole
Penicillin binding protein	43	Beta-lactams
Penicillin binding protein regulator	12	Beta-lactams
Phenicol resistance MFS efflux pumps	52	Phenicol
Polymyxin B resistance regulator	2	Cationic antimicrobial peptides
Quinolone active efflux	10	Fluoroquinolones
Quinolone resistance protein *Qnr*	173	Fluoroquinolones
Ribosomal zinc-binding protein	5	Fusidic acid
Rifampin ADP-ribosyltransferase Arr	16	Rifampin
Streptogramin A O-acetyltransferase	15	MLS
Streptogramin B ester bond cleavage	4	MLS
Sulfonamide-resistant dihydropteroate synthases	62	Sulfonamides
Sulfonamide monooxygenase	1	Sulfonamides

Tetracenomycin MFS efflux pump	1	Tetracenomycin
Tetracycline resistance MFS efflux pumps	1	Tetracyclines
Thiostrepton 23s rRNA methyltransferases	2	Thiopeptides
Tunicamycin resistance protein	1	Nucleosides
Undecaprenyl pyrophosphate phosphatase	3	Bacitracin

Table S4. Summary statistics of probe coverage. **Related to STAR Methods**

Number of target regions	4937
Target regions total length in base pairs	4803602
Expected number of base pairs covered with probes	4803581
Number of base pairs covered with probes	4793548
% of target base pairs covered with probes	99.79
Number of target sequences not covered with probes	0
Number of target base pairs not covered with probes	10054
Number of target base pairs not covered due to sequence repeats	305
% of target base pairs not covered with probes	0.21
% of target base pairs not covered due to sequence repeats	0.01
Number of probes in total	5277

Table S5. Most common sequence variants of “core” AR genes identified in fecal samples. **Related to Table 3**

Gene	Sequence variant	% samples	Sequence variant carriers from nt database*	Of those, identified in fecal samples**
ERMB	2801	91	*Streptococcus*	*Streptococcus*
CATA	1576	89	*Aeromonas salmonicida*	none
LNUC	3610	87	*Streptococcus*	*Streptococcus*
ERMF	2829	87	*Bacteroides*, *Riemerella*, *Prevotella*	*Bacteroides, Prevotella*
MSRD	4073	85	*Streptococcus, Gardnerella*, *Haemophilus*, *Neisseria*	*Streptococcus, Haemophilus*
ERMB	2799	84	*Enterococcus*	*Enterococcus*
ERMF	2833	84	*Parabacteroides*, *Bacteroides, Riemerella, Paraprevotella, Myroides, Sphingobacterium, Elizabethkingia, Alistipes, Phocaeicola*	*Parabacteroides, Bacteroides, Paraprevotella, Alistipes, Phocaeicola*
ERMB	2794	84	*Streptococcus*, *Enterococcus*, *Staphylococcus*, *Lactococcus, Bacillus*, *Clostridium, Macrococcus, Vagococcus, Anaerostipes, Ligilactobacillus, Mammaliicoccus, Campylobacter, Acinetobacter, Aliarcobacter, Vescimonas, Eggerthella*	*Streptococcus, Enterococcus, Lactococcus, Bacillus, Anaerostipes, Ligilactobacillus, Campylobacter, Eggerthella*
ERMB	2806	82	*Lactobacillus*	*Lactobacillus*

Sequence variants are provided according to the MEGARes 2.0 database structure. Only AR gene sequence variants that are present in more than 80% of samples are listed.

* — bacterial genera corresponding to sequences from nt database with 100% sequence hits to query AR gene sequence variant. Note that this column does not indicate the presence of the corresponding genera in fecal samples

** — genera identified in at least 3 fecal samples based on 16S rRNA amplicon sequencing data

Table S6. Most common sequence variants of “core” AR genes identified in oropharyngeal samples. **Related to Table 3**

Gene	Sequence variant	% samples	Sequence variant carriers from nt database*	Of those, identified in oropharyngeal samples **
MSRD	4073	99	*Streptococcus*, *Gardnerella*, *Haemophilus*, *Neisseria*	*Streptococcus, Haemophilus, Neisseria*
MEFA	3805	99	*Fusobacterium*	*Fusobacterium*
MEFA	3825	98	*Streptococcus*, *Haemophilus*, *Gardnerella*, *Gemella, Enterococcus*	*Streptococcus, Haemophilus, Gemella*
CFX	1698	97	*Bacteroides, Parabacteroides, Prevotella, Capnocytophaga, Phocaeicola, Hoylesella*	*Bacteroides, Prevotella, Capnocytophaga*
CFX	1692	94	*Capnocytophaga*, *Bacteroides*, *Parabacteroides*, *Phocaeicola*	*Capnocytophaga, Bacteroides, Phocaeicola*
CFX	1694	93	*Parabacteroides*, *Bacteroides*	*Bacteroides*
CFX	1689	93	*Prevotella*	*Prevotella*
CFX	1693	93	*Bacteroides*, *Prevotella*	*Bacteroides, Prevotella*
CFX	1697	91	*Prevotella*	*Prevotella*
CFX	1687	91	*Prevotella*	*Prevotella*
CFX	1690	91	*Prevotella*	*Prevotella*
LSAC	3647	89	*Streptococcus*	*Streptococcus*
PATA	5397	81	*Streptococcus*	*Streptococcus*

Variants are provided according to the MegaRES database structure. Only AR gene sequence variants that are present in more than 80% of samples are listed.

* — bacterial genera corresponding to sequences from nt database with 100% sequence hits to query AR gene sequence variant. Note that this column does not indicate the presence of the corresponding genera in oropharyngeal samples

** — genera identified in oropharyngeal samples based on 16S rRNA amplicon sequencing data

Table S7. The counts of common “core” gene variants present in fecal and oropharyngeal samples that were considered “biotope-specific” (adjusted p-values for Chi-square test < 0.01). **Related to Table 3**

Gene	Sequence variant	ORO smp, %	FEC smp, %	Sequence variant carriers from nt database	Of those, identified in fecal/oropharyngeal samples **
ERMB	2792	49	77	*Lactobacillus*	*Lactobacillus*
	2793	39	69	*Streptococcus*, *Enterococcus*, *Jeotgalibaca, Eubacterium, Clostridiodes, Lactobacillus, Vescimonas, Oscillibacter, Gallibacterium, Faecalibacterium*	*Streptococcus, Enterococcus, Eubacterium, Lactobacillus, Oscillibacter, Faecalibacterium*
	2794	38	84	*Enterococcus, Bacillus, Streptococcus, Staphylococcus, Clostridium, Macrococcus, Vagococcus, Butyricimonas, Ligilactobacillus, Acinetobacter, Aliarcobacter, Bacteroides, Lactococcus, Vescimonas, Mammalicoccus, Eggerthella*	*Enterococcus, Streptococcus, Butyricimonas, Ligilactobacillus, Bacteroides, Lactococcus, Eggerthella*
	2795	34	68	*Escherichia*	none
	2799	37	84	*Enterococcus*	*Enterococcus*
	2803	46	78	*Fusobacterium, Streptococcus, Enterococcus, Lactobacillus, Gallibacterium, Jeotgalibaca, Vescimonas*	*Fusobacterium, Streptococcus, Enterococcus, Lactobacillus*
	2804	49	77	*Staphylococcus, Clostridium, Enterococcus, Streptococcus, Blautia, Eubacterium*	*Enterococcus, Streptococcus, Blautia*
	2807	27	67	*Bacillus, Escherichia, Streptococcus*	*Streptococcus*
MEFA	3807	77	28	*Streptococcus*	*Streptococcus*
	3818	51	25	*Streptococcus*	*Streptococcus*
	3817	0	54	*Clostridium*	none

* — bacterial genera corresponding to sequences from nt database with 100% sequence hits to query AR gene sequence variant. Note that this column does not indicate the presence of the corresponding genera in oropharyngeal samples

** — genera identified in oropharyngeal samples based on 16S rRNA amplicon sequencing data

Table S10. DeSeq2 results of comparing AR gene abundances of samples belonging to RT1 and RT2. **Related to Figure 11**

Gene	baseMean	log2FoldChange	Adjusted p-value
QNRS	1032.284025	-5.662881	0.000000005110334895668629
IMP	505.401968	-5.340015	0.000000000992390592306639
SULI	46351.022561	-5.140814	0.000000000000000004762656
GES	7687.007181	-5.096528	0.000000000000952794960975
DFRA	2570.818035	-4.774772	0.000000000078000896610104
QACEDELTA1	1822.953684	-4.429121	0.000000045231487689131303
QEPA	123.094880	-4.233436	0.000325829608503322224776
NDM	157.340340	-3.962034	0.002013665806325893193 47
CMY	1648.648091	-3.803341	0.000224096004490388113 48
MPHA	3045.712144	-3.696569	0.000009537289304511603 13
QACE	236.680255	-3.546283	0.000224096004490388113 48
KPNE	154.836065	-3.193324	0.010632837039750899638 11
OXA	2473.568838	-2.986587	0.000000004585965129852267
OMP37	548.753650	-2.913998	0.012413903799863948082 54
CTX	73206.053635	-2.817361	0.000032861691261358134 36
KPNF	178.663346	-2.812481	0.026704488187363505102 83
PHOR	1534.084147	-2.756576	0.010732397260089406032 52
KPNO	1449.593011	-2.492181	0.001074453531885271239 87
SME	18.089999	-2.401189	0.000844184142531663765 30
KMRA	2391.099884	-2.395842	0.017138495390100212667 49
KDEA	1731.653965	-2.350539	0.019489593669722610203 01
OQXB	12012.218608	-2.298918	0.015992952751700657831 88
OQXA	3059.740328	-2.275407	0.027813479889690378987 77
AXY	7.392696	-2.143087	0.027459196809613898443 43
CARB	563.194085	-2.089127	0.020383258759008274219 05
SULII	47960.457117	-1.730265	0.012413903799863948082 54
QACAB	2.221045	-1.592889	0.048480353597885800231 99
LMRD	369.119874	-1.503795	0.004413292963427779477 81
LSAC	11.406770	1.249330	0.046961569874956171632 97

GADX	1491.523128	1.539856	0.04954186648154208089112
MDTJ	554.433619	1.833449	0.02038325875900827421905
MVRC	129.315233	3.055406	0.00014537872116387605577

Positive log2FoldChange values indicate taxa overrepresented in RT2, negative log2FoldChange values indicate taxa overrepresented in RT1, baseMean indicates expected mean.

Table S11. The results of the Wilcoxon signed rank test with continuity correction for AR genes that have shown significant changes in abundance between "Day0" and "Day7" time points for both RPKM and TPM metrics. **Related to Figure 12**

No.	gene	statistic	meandiff (RPKM)	P-value	Adjusted p-value
1	ACI	190	609.624104225719	0.000143020073393161	0.044479242825273
2	AXY	4	-27.3855262868629	8.15918593056597e-07	0.000253750682440602
3	CEOB	8	-39.6217409336698	2.03716165918574e-08	6.33557276006764e-06
4	CMX	1069	818.605399726326	3.17463875323265e-05	0.00987312652255356
5	EMRA	1095	2485.4776197159	5.24011361196257e-05	0.0162967533332036
6	EMRD	1087	6842.14705310145	1.46209952156891e-05	0.00454712951207932
7	MDTC	1033	2409.77743796569	0.000137265065605683	0.0426894354033674
8	MEXD	494	53.4130758738025	0.000141319380515872	0.0439503273404361
9	MEXI	558	92.2041346588925	7.12890278696416e-05	0.0221708876674585
10	MEXK	580	48.9534852303327	0.000107655407151339	0.0334808316240664
11	MEXQ	665	287.823474049116	2.01097229885074e-05	0.00625412384942579
12	MUXC	525	48.1901218658302	0.000104066805626927	0.0323647765499743
13	OXA	150	-9343.73387923478	1.55603189752189e-06	0.000483925920129308
14	PDC	475	173.620026301496	8.2807222250889e-05	0.0257530461200265

"Statistic” represents test statistic. "Meandiff" represents the difference of means between “Day7” and “Day0”.

Table S12. Pairs of co-abundant oropharyngeal AR genes identified as a result of Spearman correlation tests based on RPKM values. **Related to Figure 13**

**No.**	**P-value**	**Adjusted P-value**	**Rho**	**Gene 1**	**Gene 2**
1	1.363114e-151	5.410200e-148	0.9464875	MACA	MACB
2	2.811568e-118	1.115911e-114	0.9198716	MACA	NORMD
3	3.196456e-147	1.268673e-143	0.9397323	MACB	NORMD
4	9.081189e-200	3.604324e-196	0.9951210	MEFA	MSRD
5	7.394562e-41	2.934902e-37	0.9178604	MTRD	NORMD
6	7.766106e-174	3.082367e-170	0.9938914	PATA	PATB
7	5.501863e-125	2.183689e-121	0.9818547	PATA	PMRA
8	5.924928e-92	2.351604e-88	0.9390725	PATA	RLMA
9	1.819241e-117	7.220568e-114	0.9792267	PATB	PMRA
10	1.809914e-98	7.183550e-95	0.9383050	PATB	RLMA
11	1.150048e-77	4.564541e-74	0.9219260	PMRA	RLMA

Table S15. DeSeq2 results of comparing bacterial genera abundances of samples belonging to RT1 and RT2. **Related to Figure 20**

Name	baseMean	log2FoldChange	padj
CAG-352	360.63356	-3.39796	0.000000000241495
HT002	22.40025	-2.80717	0.000000000241495
Leuconostoc	33.98975	2.54808	0.000000001742023
Alloprevotella	41.72780	-2.15800	0.000021182481039
TM7x	20.01128	-1.84116	0.000042550952728
CAG-56	63.07837	1.58128	0.000644640947426
Ligilactobacillus	59.93694	-1.83203	0.000685860315463
Eggerthella	317.06928	1.66491	0.000685860315463
Adlercreutzia	9.30703	1.27568	0.001713459732047
Dialister	146.08062	-1.82753	0.001761443924259
Erysipelotrichaceae UCG-003	367.5682	1.695290	0.00315527
Weissella	26.3642	1.477956	0.00315527
Lactobacillus	63.9491	-1.368020	0.00359836
Agathobacter	997.3529	-1.434617	0.00581006
Solobacterium	24.4382	-1.209042	0.00652419
Clostridium sensu stricto 1	171.9043	-1.479549	0.00652419
Escherichia-Shigella	546.6498	1.314458	0.00882466
Lachnospira	297.6263	0.828715	0.01041431
Veillonella	102.0761	-1.142334	0.01084205
Catenibacterium	119.8207	-1.421583	0.01084205
UCG-005	393.19025	-1.058785	0.0220689
UCG-003	449.17866	1.177535	0.0289706
UCG-009	7.18051	0.883173	0.0327122
Limosilactobacillus	118.73923	-1.195831	0.0381906
Fournierella	10.46153	-0.839439	0.0381906
Anaerotruncus	7.92645	0.770558	0.0404454
Sellimonas	37.41633	1.065948	0.0481650
Barnesiella	59.63515	-1.032387	0.0481650

Positive log2FoldChange values indicate genera overrepresented in RT2, negative log2FoldChange values indicate genera overrepresented in RT1, baseMean indicates expected mean.

Table S16. DeSeq2 results of comparing bacterial species abundances of samples belonging to RT1 and RT2. **Related to Figure 20**

Name	baseMean	log2FoldChange	padj
Leuconostoc mesenteroides	20.5213	1.98554	0.0000000331541
Blautia hansenii	37.1569	1.88581	0.0000012021050
Alistipes shahii	73.2806	-1.62465	0.0000681913027
Alistipes finegoldi	65.9726	-1.55165	0.0001238045118
Bacteroides massiliensis	142.6303	1.68859	0.0002313652998
Escherichia coli	710.8258	1.39753	0.01318594386
Bacteroides ovatus	168.9707	-1.29609	0.01674711905
Streptococcus gallolyticus	14.0516	1.01783	0.01674711905
Lactobacillus gasseri	48.8872	-1.20795	0.02077969406
Streptococcus lutetiensis	21.1477	1.09977	0.02077969406
Bacteroides clarus	23.38705	-1.218571	0.0207797
Mogibacterium pumilum	5.14197	0.814291	0.0214077
Eggerthella lenta	108.29444	1.207530	0.0237778
Ruminococcus callidus	43.75722	-1.133450	0.0303618
Dialister invisus	134.81257	-1.444327	0.0337717
Akkermansia muciniphila	1148.39054	-1.435285	0.0337717
Solobacterium moorei	10.63597	0.826333	0.0378810

Positive log2FoldChange values indicate genera overrepresented in RT2, negative log2FoldChange values indicate genera overrepresented in RT1, baseMean indicates expected mean.
